# Behavior Change Techniques Implemented in Electronic Lifestyle Activity Monitors: A Systematic Content Analysis

**DOI:** 10.2196/jmir.3469

**Published:** 2014-08-15

**Authors:** Elizabeth J Lyons, Zakkoyya H Lewis, Brian G Mayrsohn, Jennifer L Rowland

**Affiliations:** ^1^The University of Texas Medical BranchInstitute for Translational SciencesGalveston, TXUnited States; ^2^The University of Texas Medical BranchCenter for Interdisciplinary Research in Women's HealthGalveston, TXUnited States; ^3^The University of Texas Medical BranchDepartment of Rehabilitation SciencesGalveston, TXUnited States; ^4^University of Central FloridaCollege of MedicineOrlando, FLUnited States; ^5^The University of Texas Medical BranchDepartment of Physical TherapyGalveston, TXUnited States

**Keywords:** electronic activity monitor, mobile, mhealth, physical activity, behavior change technique

## Abstract

**Background:**

Electronic activity monitors (such as those manufactured by Fitbit, Jawbone, and Nike) improve on standard pedometers by providing automated feedback and interactive behavior change tools via mobile device or personal computer. These monitors are commercially popular and show promise for use in public health interventions. However, little is known about the content of their feedback applications and how individual monitors may differ from one another.

**Objective:**

The purpose of this study was to describe the behavior change techniques implemented in commercially available electronic activity monitors.

**Methods:**

Electronic activity monitors (N=13) were systematically identified and tested by 3 trained coders for at least 1 week each. All monitors measured lifestyle physical activity and provided feedback via an app (computer or mobile). Coding was based on a hierarchical list of 93 behavior change techniques. Further coding of potentially effective techniques and adherence to theory-based recommendations were based on findings from meta-analyses and meta-regressions in the research literature.

**Results:**

All monitors provided tools for self-monitoring, feedback, and environmental change by definition. The next most prevalent techniques (13 out of 13 monitors) were goal-setting and emphasizing discrepancy between current and goal behavior. Review of behavioral goals, social support, social comparison, prompts/cues, rewards, and a focus on past success were found in more than half of the systems. The monitors included a range of 5-10 of 14 total techniques identified from the research literature as potentially effective. Most of the monitors included goal-setting, self-monitoring, and feedback content that closely matched recommendations from social cognitive theory.

**Conclusions:**

Electronic activity monitors contain a wide range of behavior change techniques typically used in clinical behavioral interventions. Thus, the monitors may represent a medium by which these interventions could be translated for widespread use. This technology has broad applications for use in clinical, public health, and rehabilitation settings.

## Introduction

### Background

Insufficient physical activity is a major worldwide public health problem. Even small increases in activity at a population level could have far-reaching positive impacts on chronic diseases such as diabetes, cardiovascular diseases, and several cancers [1-4]. Despite evidence supporting improved health outcomes from regular physical activity, population levels of physical activity remain low [5], and inactivity is prevalent [6].

Behavioral physical activity interventions are typically successful in increasing activity levels [7-9], but these interventions are costly and require professional expertise in delivering behavior change techniques (BCTs). Electronic activity monitors show promise as a delivery medium, as they can replicate most aspects of pedometer-based interventions while providing options for individually tailored intervention content. These monitors measure physical activity (and sometimes other health and behavior indicators such as heart rate) and interface with a computer or mobile app to provide extensive feedback tools. The feedback can be as or more rich and individualized than that provided in a clinical study, often including multiple charts, social comparisons, and indicators of progress towards individual goals. Initial intervention results using these monitors have been very promising, showing increases in physical activity and decreases in weight for two monitor brands [10-13].

The market for wearable technology activity monitors is large and growing quickly. Numerous options are currently available for use by consumers and researchers [14]. However, little is known about how these monitors differ from one another, what options they provide in their apps, and how these options may impact their effectiveness. The low cost, wide reach, and apparent effectiveness of electronic activity monitors make them appealing for recommendation by practitioners, but the growing number of options precludes practitioners’ ability to provide informed recommendations to patients. Similarly, individuals interested in using a monitor to change their behavior must rely on review websites or word of mouth to compare the variety of options. Information about the functionality of the devices and the content of their companion apps could provide guidance in choosing options most similar to standard intervention practices and best suited to individual preferences and needs.

### Behavior Change Techniques and Content Analyses

There is no consensus as to the best method for analyzing content of new media. A common method has been to use health behavior theory to create codes and/or percentage scores of the number of theoretical constructs represented. This process has been used with active video games [15], exercise apps [16], and weight loss apps [17].

A more involved method uses recently developed systematic taxonomies of BCTs to code for content that matches components of traditional behavioral interventions. Behavior change techniques are “observable, replicable, and irreducible component[s] of an intervention designed to alter or redirect causal processes that regulate behavior” [18]. A general hierarchical taxonomy of 93 BCTs has recently been published [18], and a similar taxonomy specific to physical activity and dietary interventions is also available [19]. These validated taxonomies likely provide a more informational and rigorous coding tool than previously used theory-based instruments.

In addition to describing the content of biomedical media, there is also a need to determine the extent to which the behavior change strategies are evidence-based. Several recent analyses have reviewed mobile apps using different sources for their evidence base. Sources have included the Expert Committee for Pediatric Obesity Prevention [20], the Health Education Curriculum Analysis Tool [21], best practices such as those used by the Diabetes Prevention Program [22,23], and clinical recommendations from the American Association of Diabetes Educators [24] and US Public Health Service [25,26].

Although no compendium of evidence-based best practices exists for exercise and weight loss behavioral interventions, several meta-analyses and meta-regressions have provided a general idea of the BCTs typically associated with successful change [27-29]. Investigating the prevalence of these techniques in particular may provide insight into future directions for research and development.

Further, determining how evidence-based BCTs are implemented may also improve standard coding methods. Preliminary evidence suggests that for several of the most common and effective techniques, fidelity to theory-based recommendations in their implementation enhances their effectiveness [30-32]. Thus, more in-depth analysis of implementation would provide valuable additional information, particularly for practitioners and those developing theory-based interventions.

### Content Analysis and Electronic Activity Monitors

There has been a call for study of health apps [33-35], due to their widespread use and the absence of guidelines for determining their adherence to standard practices. In addition to standalone apps, for which there are now several published content analyses [16,17,20-22,24,25], we believe that there is a need to study electronic activity monitors and their companion apps. Development of a tool for coding the content of these apps would provide valuable information for practitioners, consumers, and researchers, and could also be used to create a decision aid for determining an appropriate match of monitoring system to individual or research/clinical intervention.

The purpose of this study was to systematically investigate currently available commercial electronic activity monitors to (1) characterize their behavior change techniques, (2) determine the extent to which they include techniques associated with successful outcomes, and (3) compare implementation of several critical techniques to theory-based and evidence-based recommendations.

## Methods

### Activity Monitor Inclusion Criteria and Descriptions

Monitors were included based on three sources: review listings on CNET for wearable technology, listings in the “Health and Fitness” accessories section of the Apple Store (specifically, the Apple Web-based store that sells physical objects like iPods and MacBooks, not the online-only Apple App Store that sells apps), and a search of the Amazon site for “activity monitor”. Inclusion criteria included (1) continuous monitoring of some kind of physical activity outcome (eg, continuous measurement of steps or minutes of activity rather than discrete measurement of exercise periods), (2) the most recent iteration in a similar series of products released by the same company (eg, Fitbit Force rather than Fitbit Flex), and (3) provision of feedback via a separate mobile device or personal computer interface. Additional monitors were included based on prior knowledge or suggestion of expert colleagues if they fit inclusion criteria but were not present in the three listings above (eg, Ibitz, Lumo). Descriptions of included monitors are provided in Table 1. More in-depth descriptions of each monitor with screenshots from their Web/mobile apps can be found in Multimedia Appendix 1.

**Table 1:** Monitor names and descriptions.

Brand	Model	Where worn	Display/ compatibility	Measures	Possible measures^a^	Food/Weight tracking
Basis		Wrist	Display, personal computer, iOS^b^, Android	PA^c^, Steps, Heart rate, Skin temperature, Perspiration, Sleep		
BodyMedia	Fit	Upper arm	Personal computer, iOS, Android	PA, Steps, Sleep	Heart rate, Weight	Food, Weight, Balance
Fitbit	Force	Wrist	Display, personal computer, iOS, Android	PA, Steps, Sleep, Stairs, Distance, Calories	Weight	Food, Weight, Balance
Fitbug	Orb	Multiple	Personal computer, iOS, Android	Steps, Distance, Calories, Sleep	Weight	Food, Weight, Balance
Gruve		Waist	Personal computer	Calories, Activity zones		
Ibitz	Unity	Waist	iOS	Steps, Distance, Calories		Weight
Jawbone	Up24	Wrist	iOS, Android	PA, Steps, Sleep, SB^d^		Food, Weight, Balance
Lumo	Back	Waist	iOS, Android	Posture, Steps, Calories, Distance, SB, Sleep		
Misfit	Shine	Multiple	iOS, Android	Steps, Calories, Distance, PA, Sleep, “Points”		Food, Weight
Nike	Fuelband SE	Wrist	Display, personal computer, iOS, Android	PA, Steps, “Hours won”, Calories, “Nikefuel”		
Polar	Loop	Wrist	Display, personal computer, iOS, Android	PA, Steps, Calories, SB, Sleep	Heart rate	
Striiv	Play	Waist	Display, personal computer, iOS, Android	PA, Steps, Stairs, Distance, Calories		Weight
Withings	Pulse	Multiple	Display, personal computer, iOS, Android	PA, Steps, Sleep, Resting heart rate	Weight, Blood pressure	Weight

^a^These objective measures are tracked simultaneously by the app. Additional measurement tools must be purchased.

^b^iOS: Apple iPhone/iPad/iPod operating system.

^c^PA: physical activity.

^d^SB: sedentary behavior.

### Coding Tool and Procedure

Coding procedures for this study were based on the taxonomies of BCTs created by Michie et al [18,19]. The most recent hierarchical list was used, with published definitions guiding coding for each technique.

A tentative list of BCTs associated with successful physical activity change was created based on several recently published meta-analyses [27,28], meta-regressions [29], and systematic reviews [36-38] as well as recommendations from the US Preventive Services Task Force [39] (see Table 2).

**Table 2:** Behavior change techniques associated with physical activity change.

BCT #	Behavior change technique	Source
8.1	Prompt practice	[28,37,38]
2.3	Prompt self-monitoring of behavior	[29,36,37,39]
1.1	Goal-setting/intention formation	[29,36-39]
1.2	Barrier identification/problem solving	[38,39]
2.2	Provide feedback on performance	[29]
1.5	Prompt review of behavioral goals	[29]
5.1	Provide information on consequences of behavior in general	[27]
1.4	Action planning	[27]
10.3	Prompt rewards contingent on effort or progress towards behavior	[27,28]
6.2	Facilitate social comparison	[27,40]
4.1	Provide instruction	[27,36]
15.4	Self-talk	[39]
10.9	Self-rewards	[39]
3	Social support	[39]
7.1	Teach to use prompts/cues	[28]

Fidelity to implementation recommendations for three of the BCTs was measured based on Rovniak et al’s listing of recommendations for operationalizing mastery procedures from Social Cognitive Theory [30]. These are standard theory-based recommendations, but many of them also have demonstrated efficacy in randomized trials when compared to conditions that did not follow the recommendation(s) [30,32,41]. The full list of recommendations can be seen in the Results section.

Two trained coders (EL and ZL) wore each of the monitors for at least one 1-week period between November 11, 2013, and February 8, 2014. At least one coder wore each of the devices for 2 or more weeks. The coders downloaded and used personal computer apps and/or iPhone apps for each monitor. In cases where additional payment was required to access content (eg, a monthly subscription to use the BodyMedia device, a yearly subscription to access the full Fitbit website), we coded based on full access to all behavioral tools. Interrater reliability between the 2 coders was high (89%), with a kappa statistic of .55. An assistant coder also wore each monitor for 1 week and provided a full set of codes for each monitor. The 3 reviewers met to discuss any discrepancies, using the third coder’s results to help inform final decisions. The third set of codes informed final decisions in case of discrepancies. To update results, the 2 coders met once again in July 2014 to code 1 week’s worth of data on the monitors whose apps were updated since the previous data collection period. Coders also checked Web versions of apps where necessary. The same coding procedure was followed to determine whether additional techniques had been added.

Where functionality existed whereby a technique could be used but would not necessarily be used by default, we coded that technique as being present. For example, “friends” and “teams” are available for social support/social comparison in many apps, but users must add the friends themselves in order to take advantage of these tools. Further specific information on coder interpretation is available in Multimedia Appendix 2.

## Results

Table 3 displays the number of monitor systems found to include each BCT. Techniques from the taxonomy that were not found in any of the systems were not included in the table. The most common techniques were those that were necessarily a part of each system: self-monitoring of behavior, feedback based on that monitoring, and the addition of a monitor to the user’s environment, behavioral goal-setting, and emphasizing a discrepancy between current behavior and goal behavior. Discrepancies were typically shown via visual progress indicators, such as progress bars, pie charts, bar charts, and line charts. Charts were often color-coded to indicate proximity to the goal, which was typically set to a default of 10,000 steps per day.

**Table 3:** Behavior change techniques present in monitoring systems, by number of systems (N=13).

BCT category	BCT	Monitors, n
Goals and planning	Goal setting (behavior)^a^	13
	Problem solving^a^	1
	Goal setting (outcome)	8
	Action planning^a^	5
	Review behavior goal(s)^a^	10
	Discrepancy between current behavior and goal	13
	Review outcome goal(s)	7
	Commitment	4
Feedback and monitoring	Feedback on behavior^a^	13
	Self-monitoring of behavior^a^	13
	Self-monitoring of outcome(s) of behavior	8
	Biofeedback	2
	Feedback on outcome(s) of behavior	8
Social support	Social support (unspecified)^a^	8
	Social support (practical)	2
	Social support (emotional)	4
Shaping knowledge	Instruction on how to perform the behavior^a^	2
	Information about antecedents	1
Natural consequences	Information about health consequences^a^	6
	Information about social and environmental consequences^a^	1
	Monitoring of emotional consequences	4
	Information about emotional consequences^a^	1
Comparison of behavior	Social comparison^a^	8
Associations	Prompts/cues	7
Repetition and substitution	Behavior substitution	1
	Habit formation	1
	Graded tasks	3
Comparison of outcomes	Credible source	2
Reward and threat	Non-specific reward	6
	Social reward	8
	Reward (outcome)	1
Antecedents	Adding objects to the environment	13
Scheduled consequences	Situation-specific reward	3
	Reward incompatible behavior	1
Self-belief	Focus on past successes	7

^a^This BCT was identified in the literature as associated with successful intervention.

Six techniques were present in half or more of the monitoring systems. Reviewing behavioral goals (10/13 systems) was coded when systems allowed and/or encouraged users to adjust their goals over time. Social support, social comparison, and social reward were also common (8/13 systems). Tools that allowed social support included friending systems and groups, commenting and emoticon systems for communication with others, and the ability to exercise with others virtually in real time. Social comparison was typically found in the form of lists (leaderboards), charts, and direct statements of comparison to other users. Social rewards consisted primarily of opportunities to share accomplishments and progress via social networks. Prompts or cues were found in seven systems. These were typically inactivity or idle alerts. Systems that alerted via a monitor used vibration or flashing lights to attract attention, while those that alerted via mobile device used push notifications. Seven systems also demonstrated a focus on past success, operationalized here as weekly/monthly/yearly emails discussing progress towards goals. Other techniques were found in fewer than half of the systems.

**Figure 1:**
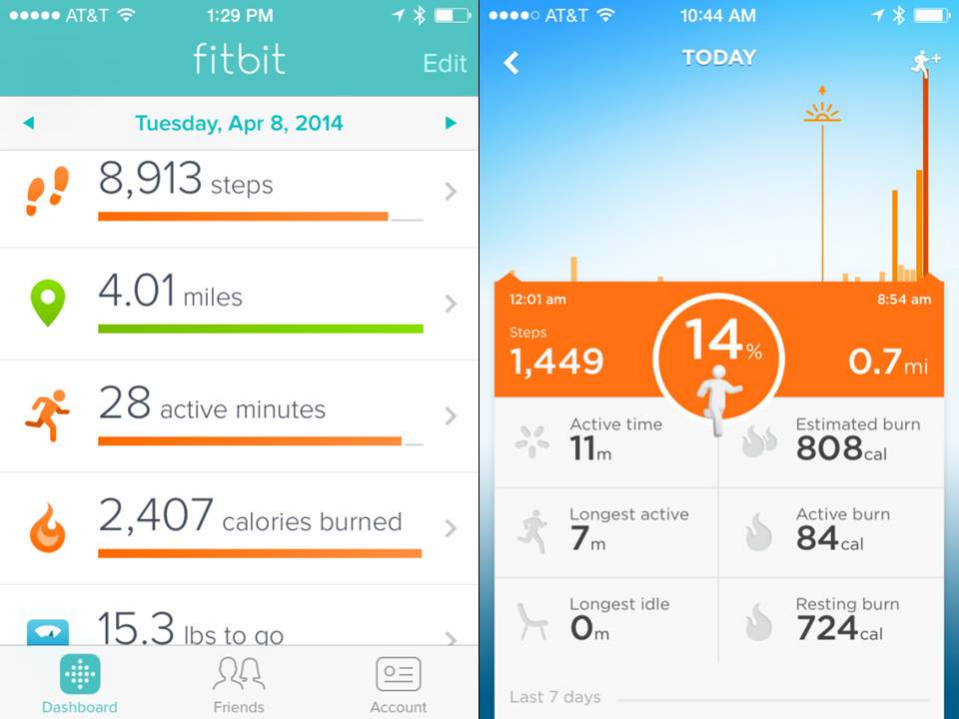
Example screen shots from Fitbit and Jawbone.

Figure 1 displays examples of screens from the Fitbit (left) and Jawbone (right) apps. See Multimedia Appendix 1 for further examples of the BCTs discussed below, each taken from one of the studied Web/mobile apps. The full listing of BCTs found in each monitoring system is presented in Multimedia Appendix 2.

Several of the more common techniques were among those found in the literature to be associated with physical activity (shown broken down by monitor system in Multimedia Appendix 3). Goal-setting, self-monitoring, and feedback were found in all of the systems. Social comparison, review of behavioral goals, social support, and social rewards were present in more than half of the monitoring systems. However, several techniques associated with successful interventions were less common. Information about consequences of the behavior and non-specific rewards were each found in six systems. Instruction on performance of the behavior, action planning, and problem solving were rare. Prompting practice, self-rewards, and self-talk were not found.

Table 4 displays theory-based recommendations for goal-setting, self-monitoring, and feedback. Overall, these recommendations were mostly followed by most of the systems. The recommendations less likely to be followed included breaking long-term goals into short-term goals (few systems included both types of goal), progression from easier to more difficult goals, tracking personally valued information, emphasizing performance successes, and comparing performance to norms of similar groups.

**Table 4:** Fidelity of monitoring systems to recommendations for goal-setting, self-monitoring, and feedback.

Technique	Recommendation	Monitors, n
Goal-setting	Specific	13
	Measurable	13
	Moderately challenging	13
	Long-term goals broken into short-term goals	6
	Easier goals successfully accomplished before attempting more difficult ones	3
Self-monitoring	Conducted regularly	13
	Conducted close in time to target activity	13
	Track precise information	13
	Track personally valued information	6
	Emphasize performance successes	9
	Focus on behavior modifiable by deliberate effort	13
Feedback	Specific	13
	Give a clear idea of how well participant is doing	13
	Compare performance to past accomplishments	13
	Compare performance to norms of similar groups	5
	Compare performance to precise goals	13

## Discussion

### Principal Findings

Electronic activity monitor systems include a variety of evidence-based BCTs, many of which conform to recommendations for their implementation. The most commonly found techniques were integral to the nature of the monitor: self-monitoring, feedback provision, adding objects to the environment, and goal-setting. Tools that provided or encouraged review of behavioral goals, social support, social comparison, prompts/cues, rewards, and a focus on past success were also common, found in more than half of the systems. Most of the interactive tools for goal-setting, self-monitoring, and feedback conformed to theory-based recommendations. Unfortunately, several techniques associated with successful physical activity intervention were uncommon or absent from the monitor systems, including practice, action planning, and problem solving.

### Behavior Change Techniques and eHealth/mHealth

Several recently published articles have provided an overview of the current state of mobile apps for physical activity and weight loss. The results suggest that most apps do not include many BCTs that are thought to be essential to behavioral intervention. For example, one study found that 28% of pediatric obesity prevention apps included goal-setting [20], and a broader study of physical activity apps found that 44% included monitoring of some kind [21]. A study that compared weight loss apps to components of the widely used and validated Diabetes Prevention Program reported better results, with 90% or more including weight loss goals and dietary goals. However, only 20% included a physical activity goal, and fewer than 5% included problem-solving or habit formation [22]. A sample of diabetes apps were found to include a median of only two out of seven self-management behaviors recommended by the American Association of Diabetes Educators [24]. An analysis of fitness video games found greater percentages that included these techniques, such as feedback (17/18), rewards of some kind (16/18), and practice (15/18) [15]. Two content analyses of physical activity apps that used taxonomies of 23 and 26 BCTs, respectively, found that the apps included an average of 5/23 and 8/26 [42,43].

Of the activity monitor apps analyzed here, all 13 included monitoring and goal-setting. Although weight loss was not the primary purpose of most of these systems, 62% (8 /13) included weight loss goals. The prevalence of problem solving (1/13) and habit formation (1/13) was similar to that found in the other apps. As might be expected by the nature of fitness video games versus activity monitor apps, practice was much more prevalent in the video games than in the monitor systems. Rewards were fairly common in the monitor systems (6/13), but not as common as in true video games. These rewards were typical of “gamified” reward systems, including badges and achievements.

The three systems with the most techniques coded (Jawbone, Fitbit, and Nike) included 27, 20, and 19 techniques, respectively, out of the 93 possible. The absolute number of techniques found in a monitoring system may not be informative; in fact, a system with fewer but more effective techniques may ultimately produce a greater impact than a system with more numerous but less effective ones. Further, there exist several iterations of the behavior change taxonomy, ranging from 26 techniques to 93. The total number of possible techniques to be coded makes comparison across studies difficult. A recent meta-analysis of walking and cycling interventions reported that the interventions studied included a mean of approximately six BCTs, ranging from 0-12 out of a possible 26 [36]. A similar study of physical activity interventions among overweight/obese adults found 3-12 techniques out of a possible 40 [38]. The number of techniques found in the monitoring systems studied here ranged from 9-27 out of 93, with most including 12 or more. Upon recalculation using only those techniques that also exist in the 40-item taxonomy [19], we found that the activity monitor systems included 6-12 out of 40 techniques, with an average of 9 techniques per system. Recalculation using the original 26-item taxonomy found an average of 8 techniques out of 26 (range 6-12). Thus, although exact comparison is impossible due to differences in taxonomies over time, it appears that the monitors include a similar number of techniques as can be found in behavioral interventions and a potentially greater number of techniques than found in standard physical activity mobile apps.

Across multiple meta-analyses of physical activity interventions, several techniques were reported to occur in more than half of studied interventions: self-monitoring of behavior, goal-setting, providing instruction, problem solving, and prompting practice [27,28,36,44]. Self-monitoring and goal-setting were also common among the monitoring systems, but instruction, problem solving, and practice were very uncommon. Though the basic content of physical activity interventions and activity monitor systems (self-regulatory techniques such as self-monitoring, goal-setting, and feedback) are the same, monitor systems differ greatly from traditional interventions in the other implemented techniques.

### Adherence to Theoretical and Empirical Best Practices

Of the 14 BCTs identified as potentially effective based on their success in previous interventions, five were widely represented across the devices: goal-setting (behavior), review of behavioral goals, feedback of behavior, self-monitoring of behavior, and rewards. Problem solving, action planning, commitment, instruction on how to perform the behavior, and behavioral practice were rare. It may be that these less common techniques are not prioritized by developers or consumers, or perhaps they are more difficult to implement. Problem solving was found in one app, but it provided only generalized tips to overcome problems (some specific to detected behavior and some discussed as common problems). Individualized problem solving would likely be a complex undertaking that would require self-report of barriers and a system for providing automated counseling. Such a system might increase app size unacceptably or be difficult to program. Action planning and commitment occurred in the context of specific challenges that users pledged or committed to undertaking. Gamification such as this appears to be a promising avenue for implementing less common BCTs.

A recent study used classification and regression trees to statistically investigate the effectiveness of combinations of BCTs [45]. The investigators found that a combination of techniques that are now called goal-setting and providing information about consequences was most successful (the analysis used a previous iteration of this taxonomy with slightly different names). They also found that interventions using feedback provision in the absence of review of behavioral goals or information about consequences were the least effective of those studied. Self-monitoring and feedback provision are the backbone of monitor systems, but many systems do not include any kind of information provision regarding specific consequences of behavior. It may be that these bare-bones apps that focus on function do not provide sufficient motivation to encourage consistent activity over time.

Several previous studies have scored apps based on their adherence to theoretical constructs. For a general study of several types of health apps, the mean score found was approximately 8/100, with the highest-scored app receiving 14/100 [17]. For a similar study specific to physical activity apps, the mean score was approximately 10/100, with the highest-scored app rated 28 [16]. It would appear that the activity monitor apps included in this analysis follow theory (here, specifically Social Cognitive Theory) more closely than apps that are not associated with activity monitors. It may be that activity monitors by definition provide behavioral tools that are suggested by Social Cognitive Theory, such as regular, instant, and precise feedback.

The above information leads to the question of what the ideal monitor and monitoring system might include. It is not surprising that two of the most well-known and popular monitors, from Fitbit and Jawbone, were highly adherent to theoretical principles (Fitbit) and evidence-based principles (Jawbone). The Jawbone Up24 was particularly impressive for including all but one of the best practice techniques investigated. However, despite their utility in previous clinical and community interventions, we do not yet know whether these techniques work well in concert and in the context of a wearable device and monitoring app.

Regardless of the number or effectiveness of BCTs included, success for an individual is likely highly influenced by individual preferences and practical issues. For example, the Misfit Shine is the only waterproof monitor of those tested and thus would likely be the most effective for someone who prefers to swim. The BodyMedia, Fitbit, Fitbug, and Jawbone systems provided energy balance information including food logs, which may make them more suitable for weight loss attempts than systems that monitored only activity and weight (although several other monitors can link to other apps that provide this service). Little is known about the reliability and validity of these devices, which could also influence user preferences. Because of the complicated series of variables that potentially influence effectiveness, a decision aid similar to those used in patient-centered outcomes research would be a logical next step for helping potential users choose a monitor in light of their preferences for techniques, game and social functions, appearance, and usability.

### Clinical Applications

There exists a large and growing amount of literature demonstrating the utility of Internet- and technology-enhanced (generally called eHealth) energy balance interventions. Although several reviews have found that computer-mediated or telephone-mediated weight loss interventions were less powerful than traditional face-to-face interventions [46,47], a recent meta-regression did not find a significant effect of in-person contact on weight loss at 12 months [40]. Thus, it is currently unclear whether technology-mediated interventions can consistently replicate the effectiveness of standard clinical interventions. Activity monitor apps by definition include self-monitoring and individualized feedback, which are associated with greater effectiveness in technology-based trials [46]. These monitors may be a medium by which more effective tools can be integrated into self-directed, distance interventions.

Although little is known about the efficacy of electronic activity monitors, several clinical trials have provided preliminary data. To our knowledge, three trials have tested BodyMedia’s SenseWear armband, a clinical/research grade armband that is very similar to the commercially available BodyMedia Fit armband. An early study found that adding continuous use of the armband to a 12-week standard behavioral weight loss program produced additional weight loss of approximately 2 kilograms [10]. This finding was not statistically significant in this small sample, but it may be clinically significant if distributed over a large population with respect to disease prevention and health cost reduction. A later study compared a 6-month standard behavioral weight loss (SBWL) program, SBWL plus the armband system, and the armband system alone and found a 5-kilogram difference between SBWL plus armband and SBWL [12]. A 9-month study found a 3-kilogram difference between a SBWL and SBWL plus armband group; however, this difference was not statistically significant [11].

Beyond these more typical implementations for clinical weight loss interventions, electronic activity monitors may also be a useful measure of patient-reported outcomes. Some researchers have begun using patterns of patient ambulation during and after hospitalization as a proxy measure for health, as these patterns can predict readmission [48] and other health outcomes such as quality of life and functional status [49]. Consistent, objective measures provided by these monitors could allow clinicians to identify at-risk individuals for secondary prevention and rehabilitation interventions. The CYCORE (Cyberinfrastructure for Comparative Effectiveness Research) project has demonstrated initial feasibility and acceptability of a system of home-based sensors, including activity monitors, that transmit information to oncologists for early detection of dehydration among head and neck cancer patients [50]. They could also be used to help determine appropriate lengths for hospital stays and to monitor functional independence post-release [51].

Health care professionals’ preferences likely will play a role in how successful a given system is for users who are prescribed the device. Physicians, interventionists, and counselors may find that some of the companion apps are easier to integrate into their personal approach to patient care than others. Ease of surveillance may also play a role in provider choice of monitors. Some monitors more easily lend themselves to various types of surveillance, either by allowing “friends” to view user data, by partnering with other apps that allow for practitioner or friend surveillance, or by allowing users to export their data to third parties. Official methods of transmitting data to practitioners securely do not appear to currently exist in these apps. However, upcoming health information aggregator apps like those made by Apple and Google may provide a method for automatically updating physicians in the future.

### Public Health and Community Applications

From a public health perspective, electronic activity monitors hold promise for large-scale, cost-effective activity and energy balance interventions. Much like previous studies of Internet-based behavioral weight loss interventions, monitor-based interventions may be less powerful than standard face-to-face programs [10,12,52]. However, they may also have a greater public health impact due to greater reach, adoption, implementation, and/or maintenance [53]. Initial investigations of the BodyMedia armband have found that it provides a more cost-effective weight loss intervention than standard behavioral weight loss interventions or combinations of the two [52].

Some of the monitors demonstrated a greater emphasis on energy balance, providing tools for monitoring intake, comparing intake to expenditure, and monitoring weight loss (eg, BodyMedia, Fitbit, Fitbug). The apps for BodyMedia, Fitbit, Fitbug, and Withings communicated with smart scales, which automatically uploaded weight measurements to the apps. These monitors and their apps provide interactive tools that mimic a large proportion of the techniques of behavioral weight loss interventions that require skilled interventionist time. These tools could reduce the time needed by interventionists for counseling by creating automated feedback.

Several of the monitoring systems included measurement and cues related to sedentary behavior. Preliminary studies that provided feedback based on baseline analyses of sedentary behavior (using research-grade monitors) produced promising results [54,55]. The existence of commercial monitors that can provide continuous real-time feedback related to sedentary behavior as well as physical activity increases the options available to interventionists. Lumo (sit time, stand ups), Polar (resting, sitting, and low intensities), and Jawbone (longest idle period) monitors measured sedentary behavior and provided mobile phone reminder alerts when sedentary periods extended past a pre-set threshold. These monitors and others that adopt this functionality could be used to implement larger-scale and lower-cost sedentary behavior interventions than those in the past [53].

### Rehabilitation Applications

Electronic activity monitors have the potential to significantly improve objective measurement of physical activity for people with chronic diseases and disabilities who receive physical therapy, occupational therapy, and other types of rehabilitation services. While much has been written about the use of pedometers [56,57], accelerometers [56,58,59], and self-report questionnaires [59,60] to measure physical activity for rehabilitation patients, very little has been published expanding to other types of physical activity measurement for these populations. One exception is the development of wearable sensors, such as those described by Bonato et al [61]; however, widespread adoption and testing of wearable technology devices is not evident in the peer-reviewed literature. However, there is agreement among researchers that an effective means of quantifying physical activity is needed. Electronic activity monitors have the potential to offer a solution for gaps in current monitoring systems. For example, these monitors offer researchers and consumers the opportunity to gather physical activity data in real-world conditions such as home and community settings. They also have the capacity to provide real-world behavioral motivation using prompts and intensity measures that are variable or absent in current monitoring methods. Talkowski et al have pointed out the need for accurate physical activity intensity measures that are not currently being accurately evaluated [60]. These authors note that the number of hours of therapy is often a proxy for estimating the intensity of a rehabilitation program, whereas the length of time in therapy may not offer a uniform intensity across patients and over time. An electronic physical activity monitor would provide an objective measure of treatment intensity.

### Potential for Unintended Consequences

Though numerous positive applications of these electronic activity monitors exist, there is always the possibility for unintended adverse consequences or ethical dilemmas. The potential for sharing of global positioning system (GPS) location data and personal health information produces clear privacy concerns. Surveillance of the collected data by health care providers may also lead to situations where intervention is deemed ethically necessary. Clear protocols will be necessary to guide provider behavior in such cases and to reduce risks associated with potential privacy breaches.

Because these monitors are commercially available, they can be used by individuals without consultation with medical or public health professionals. Although this widespread availability has benefits for accessibility, it might also increase the risk of negative outcomes if potentially dangerous activity programs are begun without professional oversight. The default activity goals may be inappropriate for older adults, individuals with disabilities or chronic conditions, or children [62]. Though some apps allow users to change their goals, or set goals for them based on a baseline measurement period, others provide pre-set goals that cannot be adjusted. These goals may provoke inappropriately intense activity that could lead to injury.

The validity and reliability of these monitors’ step estimates is as yet unclear. Substantial literature surrounding the validity of the research grade BodyMedia armband exists (eg, [63,64]), but it is not clear whether differences between the research and commercial versions may affect energy expenditure estimates. There is preliminary evidence that one of the Fitbit monitors (worn on the waist) may produce valid estimates of steps, but distance output is inaccurate [65]. Another study found that older Fitbit monitors underestimated energy expenditure [66]. Little is known about newer, wrist-worn monitors or how monitors may differ (both from other commercial monitors and compared to gold standard measures).

### Limitations

As a content analysis, this project was by definition preliminary and exploratory. Thus, our conclusions are tentative and require further study. In particular, our coding related to theory-based recommendations and our designation of specific techniques as potentially more effective than others are intended to be first steps towards formal tests as to the true impact of various recommendations or techniques. Only research with human subjects—from small qualitative investigations to large-scale randomized trials—can investigate hypotheses related to feasibility, acceptability, and effectiveness.

The systems tested here were those that measure continuous lifestyle activity that were available for purchase in late 2013. Monitors that had been discontinued (eg, Motorola MOTOACTV and Larklife) could not be tested, nor could a large number of monitors expected to be released in 2014. Follow-up tests should be conducted to include these newer monitors and compare them to earlier models. Also, we did not include monitors that focused specifically on bouts of physical activity (eg, heart rate monitors by Garmin, Polar, Mio) or mobile phone apps that measured activity using GPS or accelerometry within the phone (eg, RunKeeper, phone-based pedometer apps). Our focus on products compatible with Apple iOS, which occurred for practical reasons, may have also led to missing some monitors only available for Android devices if they were not also listed on Amazon or CNET lists. To represent the full range of available options and for use in possible future decision aids, further testing of all these monitoring systems will be necessary.

We chose to use the latest and broadest taxonomy available, which likely contributed to the greater number of techniques found in these systems. Many of the techniques in the larger taxonomy are not used in physical activity intervention (eg, many of the associations techniques are more appropriate for addiction-related interventions) and likely should not be included in activity monitor apps. It is also possible that some techniques are counterproductive or only productive in conjunction with specific other techniques. Even otherwise appropriate techniques, such as behavioral practice, may be unnecessary when the activity being promoted is an activity of daily living like walking. Which techniques are most efficacious is, of course, an empirical question not yet answered.

Finally, coding of ever-changing apps is quite difficult. Some interrater disagreement occurred because only one of the 3 testers engaged in a behavior that triggered use of a specific technique. As all monitors were tested using personal computers and iOS mobile devices, the experiences of Android users may differ from our experiences. Regular app updates also led to differential coding. Although we updated our results prior to publication, it is likely that more techniques will be included across the 13 systems and new systems will be available in the near future.

### Conclusions

Electronic activity monitors include many different empirically tested behavior change techniques that are commonly implemented in clinical interventions. Many of these techniques are associated with successful physical activity and/or weight loss, and implementation of most of the techniques adhered closely to theory-based recommendations.

This content analysis provides preliminary information on the extent and type of technique implementation, thus laying a foundation for clinical, public health, and rehabilitation applications. Future studies are needed to further investigate new types of electronic activity monitors and to test their feasibility, acceptability, and ultimately their public health impact.

## Multimedia Appendix 1

Screenshot examples of behavior change techniques.

## Multimedia Appendix 2

Behavior change techniques by monitor.

## Multimedia Appendix 3

Potentially effective techniques by monitor system.

## Abbreviations

BCTs: behavior change techniques
GPS: global positioning systems
iOS: Apple iPhone/iPad/iPod operating system
SBWL: standard behavioral weight loss
