# Supplementary material for: Behavior Change Techniques Implemented in Electronic Lifestyle Activity Monitors: A Systematic Content Analysis
Source: J Med Internet Res. 2014 Aug 15;16(8):e192. doi: 10.2196/jmir.3469 (PMC4147713; doi:10.2196/jmir.3469)
Supplement: Supplementary file 1 [file jmir_v16i8e192_app1.pdf]

Multimedia appendix 1. Screenshot examples of behavior change techniques

Note: some screenshots may differ from others of the same app due to changes in appearance and functionality over time. Many of these apps were updated regularly.

1. Goals and planning

1.1. Goal setting (behavior)

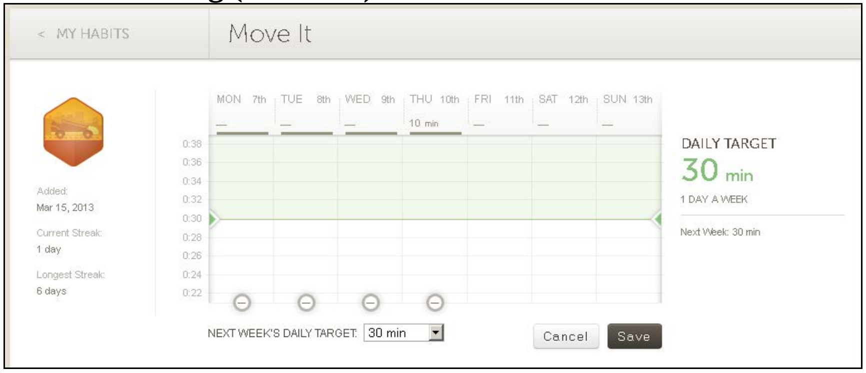

Basis (PC)

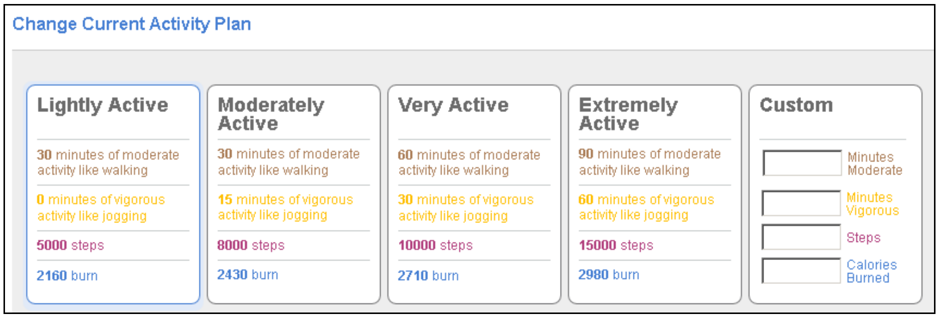

BodyMedia (PC)

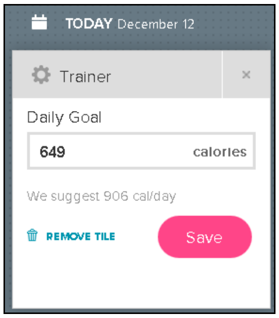

Fitbit (PC)

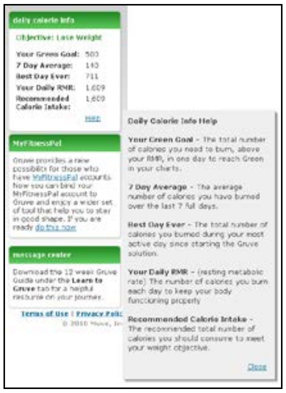

Gruve (PC)

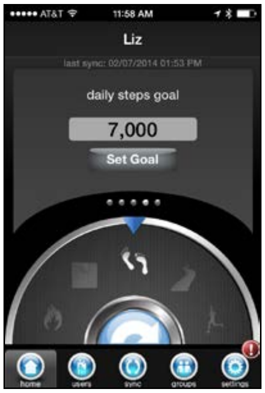

Ibitz (iOS)

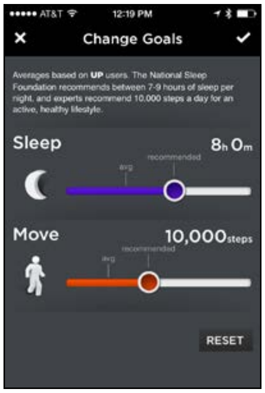

Jawbone (iOS)

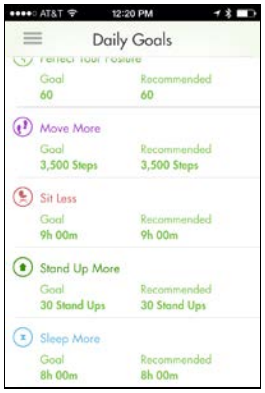

Lumo (iOS)

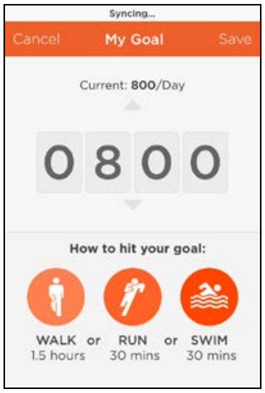

Misfit (iOS)

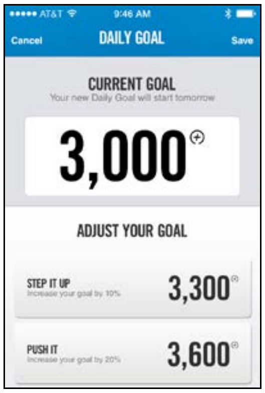

Nike+ (iOS)

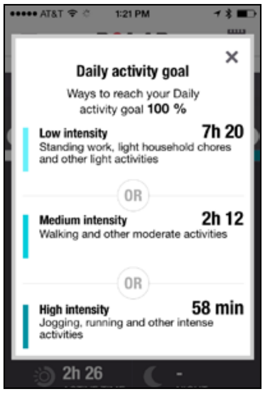

Polar (iOS)

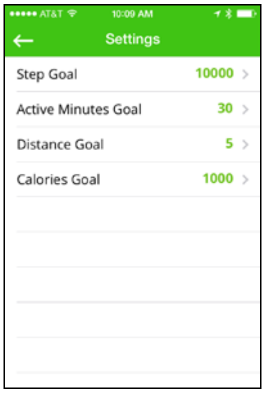

Striiv (iOS)

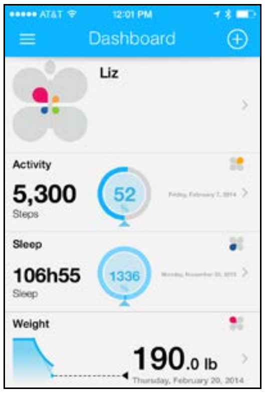

Withings (iOS)

1.2. Problem solving

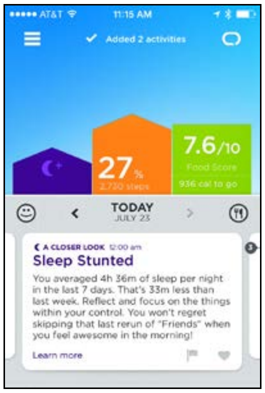

Jawbone (iOS)

1.3. Goal setting (outcome)

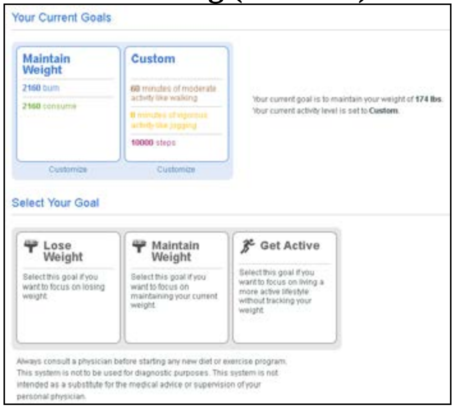

BodyMedia (PC)

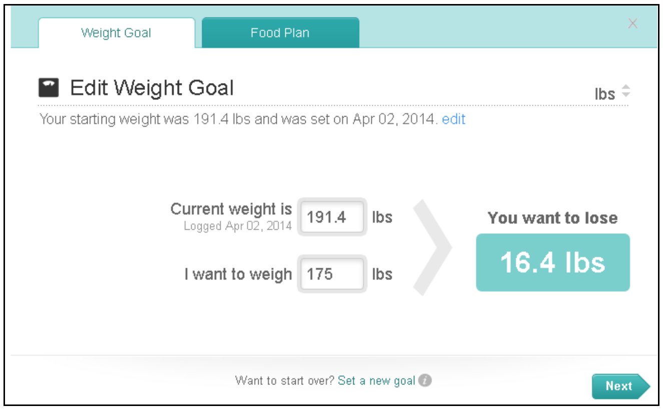

Fitbit (PC)

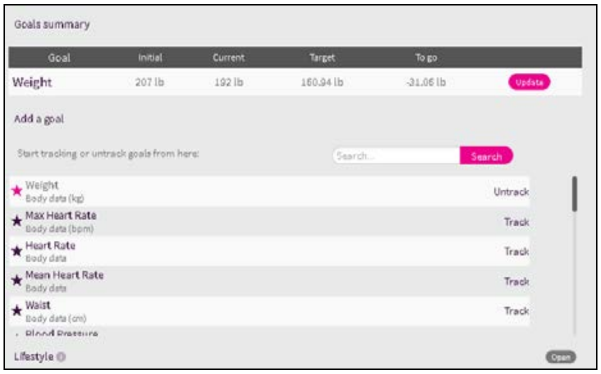

Fitbug (PC)

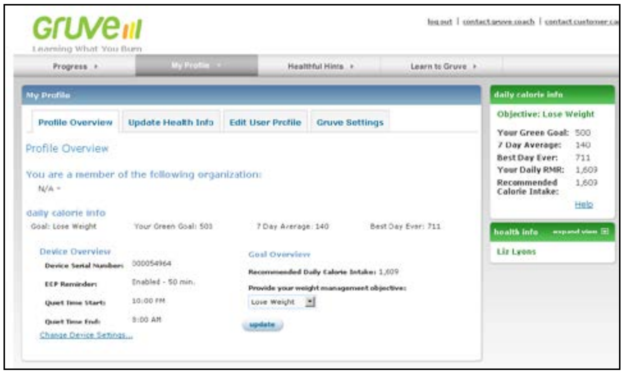

Gruve (PC)

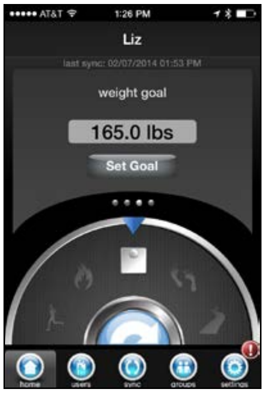

Ibitz (iOS)

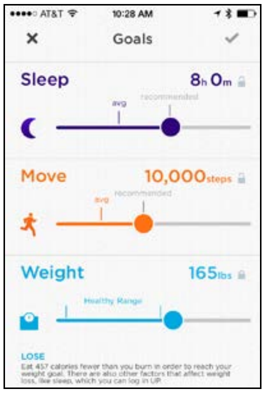

Jawbone (iOS)

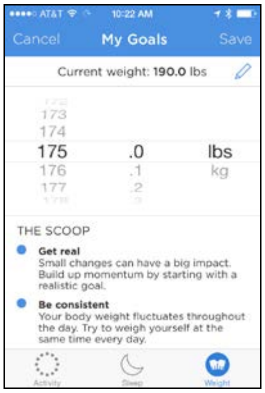

Misfit (iOS)

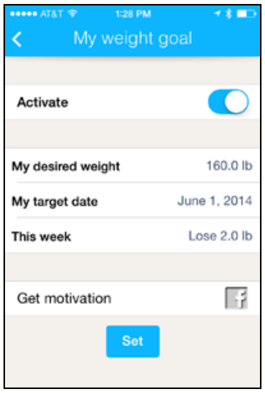

Withings (iOS)

1.4. Action planning

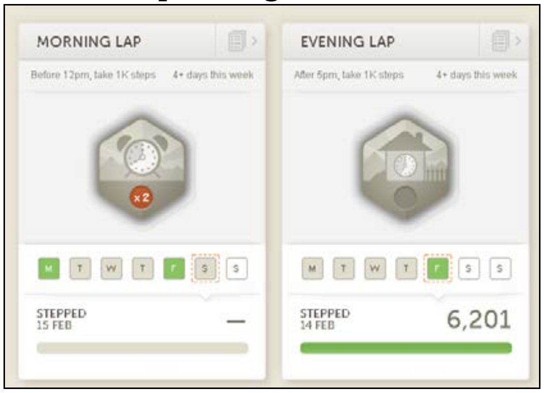

Basis (PC)

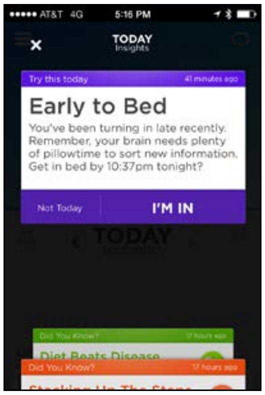

Jawbone (iOS)

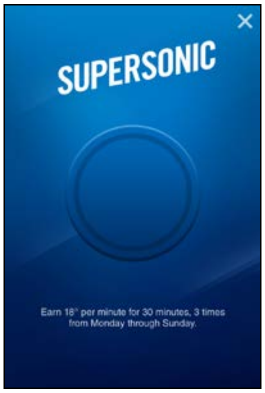

Nike+ (iOS)

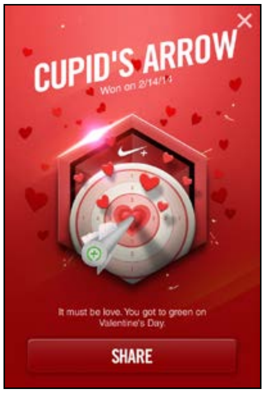

Nike+ (iOS)

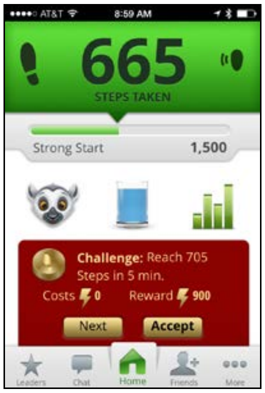

Striiv Walkathon(iOS)

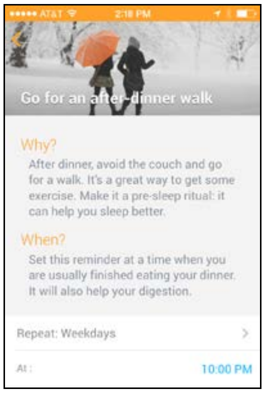

Withings (iOS)

1.5. Review behavior goal(s)

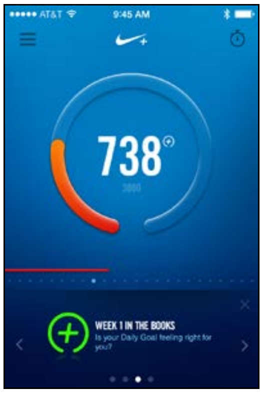

Nike+ (iOS)

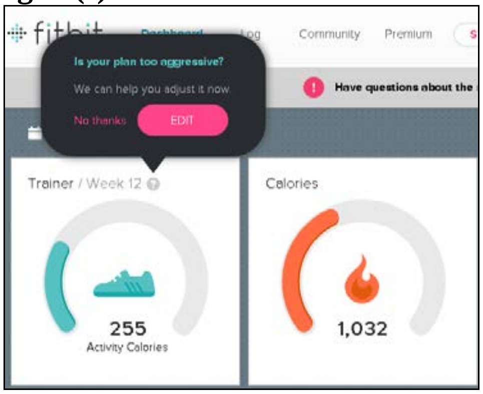

Fitbit (PC)

See 1.1, Goal setting (behavior) for more examples

1.6. Discrepancy between current behavior and goal

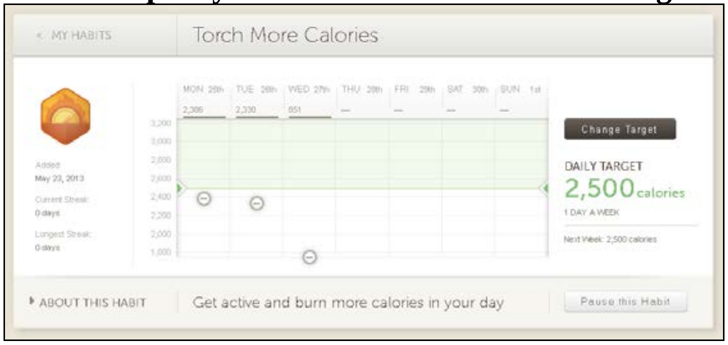

Basis (PC)

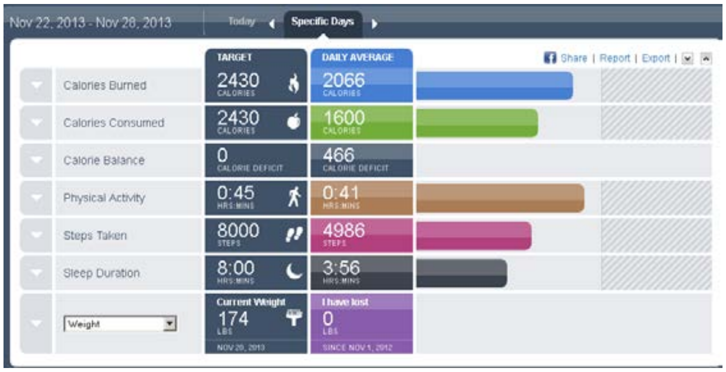

BodyMedia (PC)

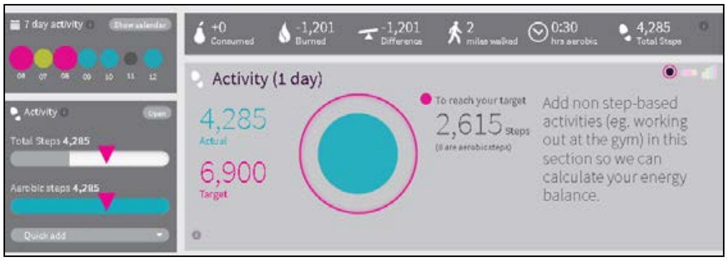

Fibug (PC)

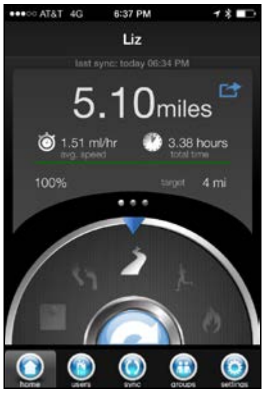

Ibitz (iOS)

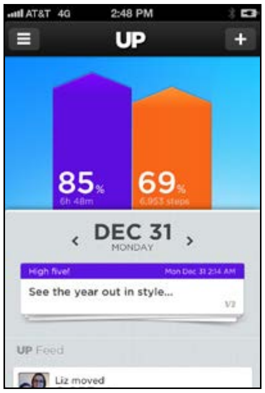

Jawbone (iOS)

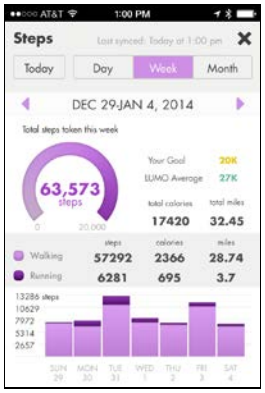

Lumo (iOS)

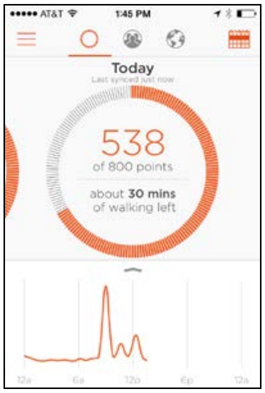

Misfit (iOS)

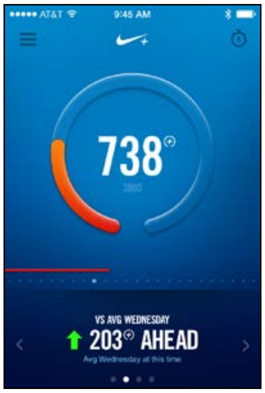

Nike+ (iOS)

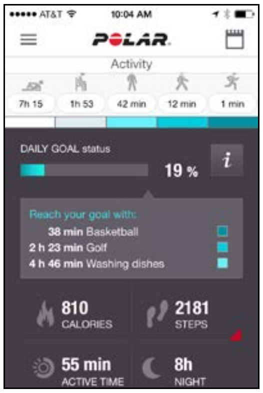

Polar (iOS)

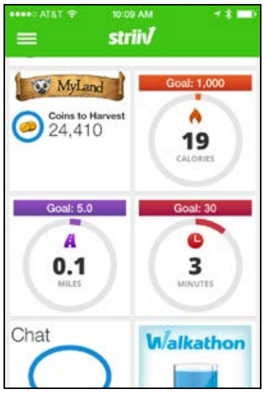

Striiv (iOS)

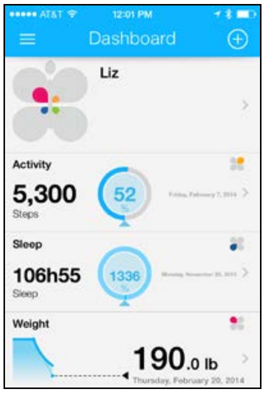

Withings (iOS)

1.7. Review outcome goal(s)

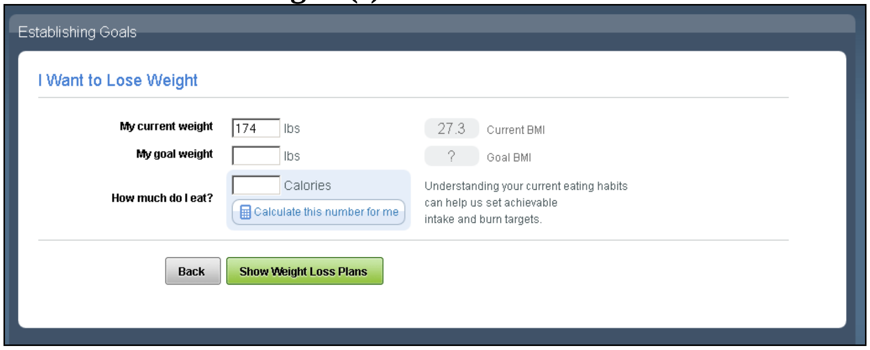

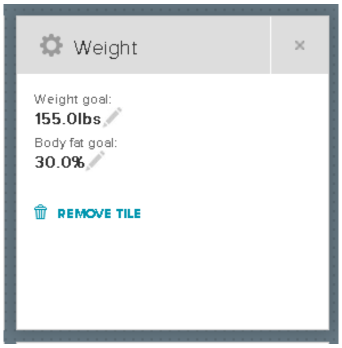

BodyMedia (PC)

Fitbit (PC)

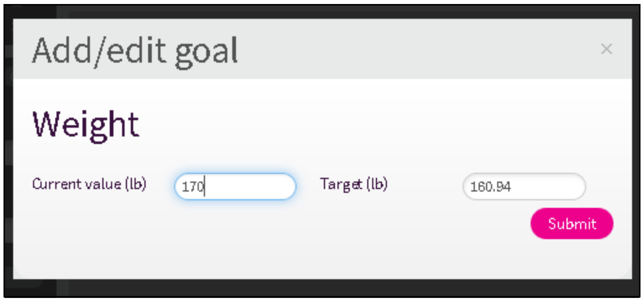

Fitbug (PC)

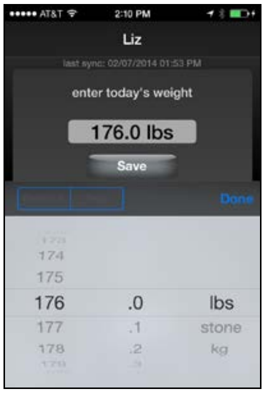

Ibitz (iOS)

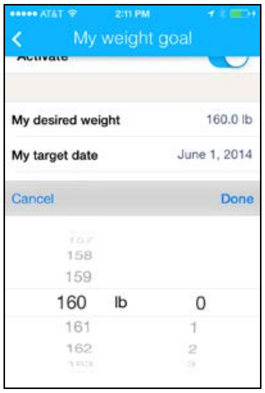

Withings (iOS)

1.9 Commitment

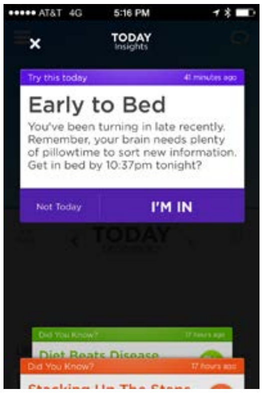

Jawbone (iOS)

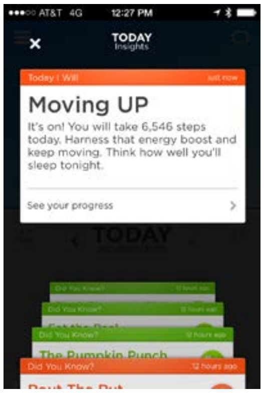

Jawbone (iOS)

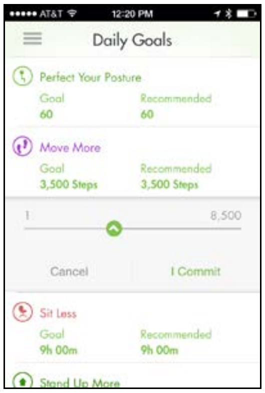

Lumo (iOS)

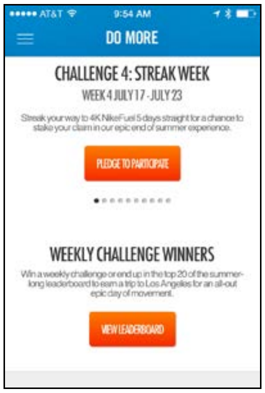

Nike+ (iOS)

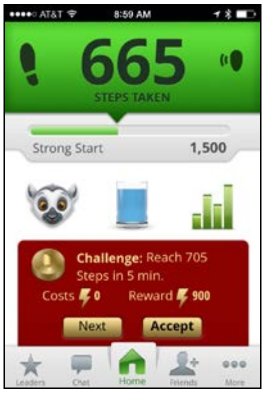

Striiv Walkathon(iOS)

2. Feedback and monitoring

2.2. Feedback on behavior

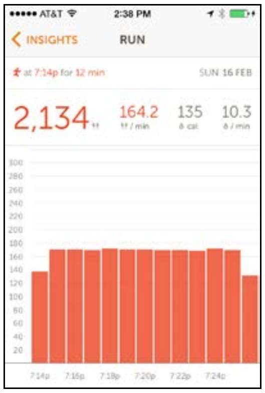

Basis (iOS)

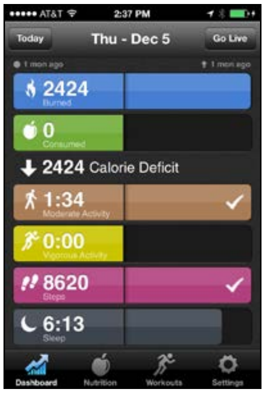

BodyMedia (iOS)

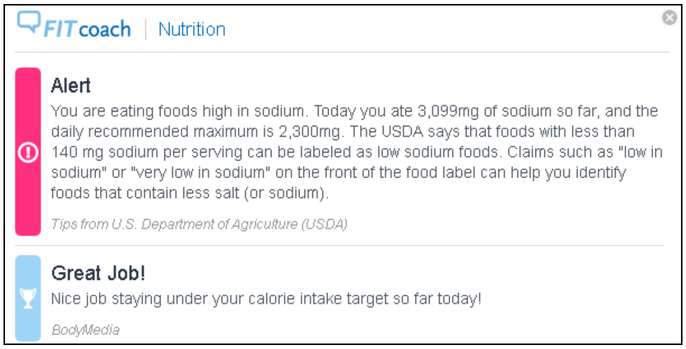

BodyMedia (PC)

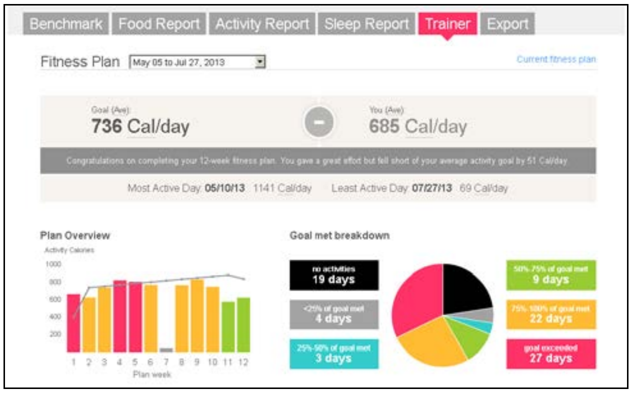

Fibit (PC)

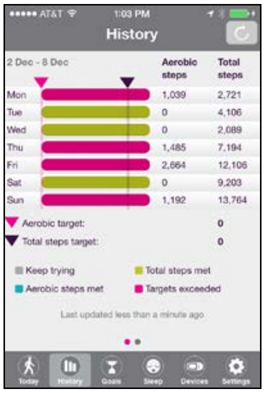

Fitbug (iOS)

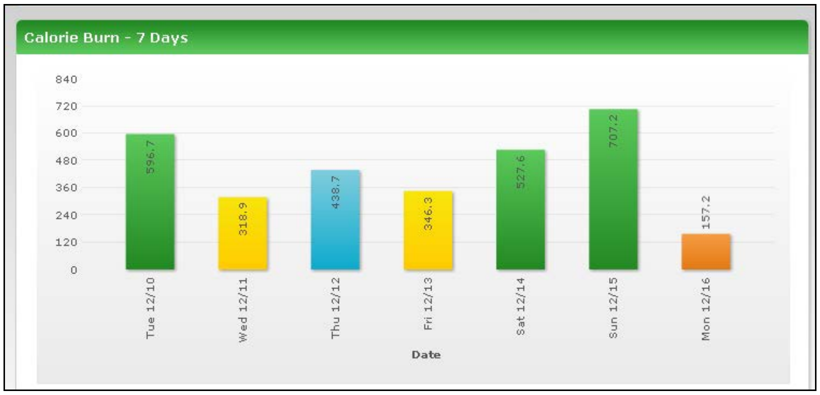

Gruve (PC)

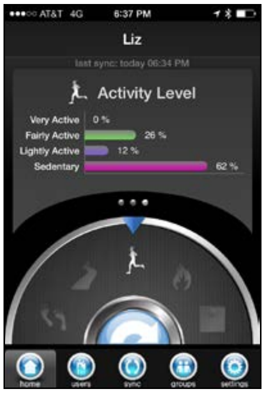

Ibitz (iOS)

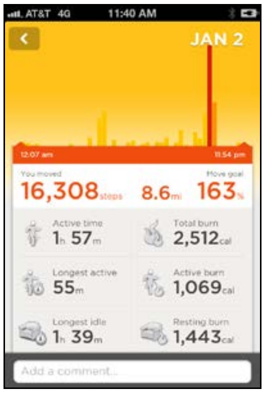

Jawbone (iOS)

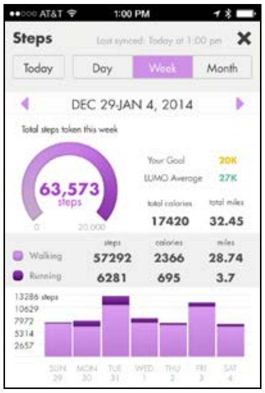

Lumo (iOS)

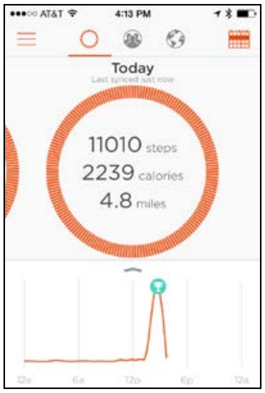

Misfit (iOS)

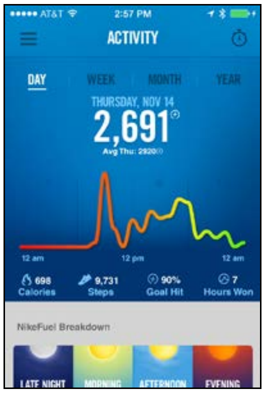

Nike+ (iOS)

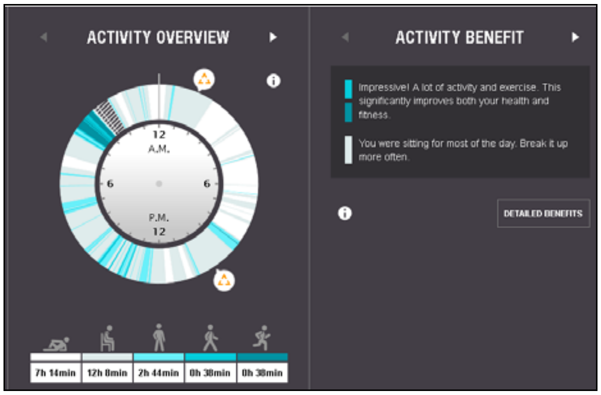

Polar (PC)

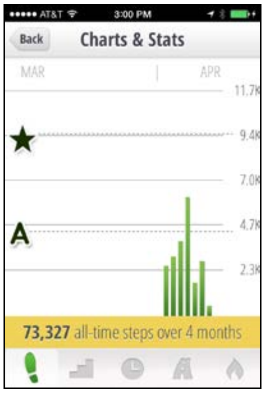

Striiv Walkathon(iOS)

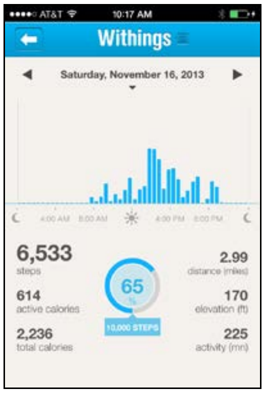

Withings (iOS)

2.3. Self-monitoring of behavior

See sections on feedback for examples

2.4. Self-monitoring of outcome(s) of behavior

See sections on feedback for examples

2.6. Biofeedback

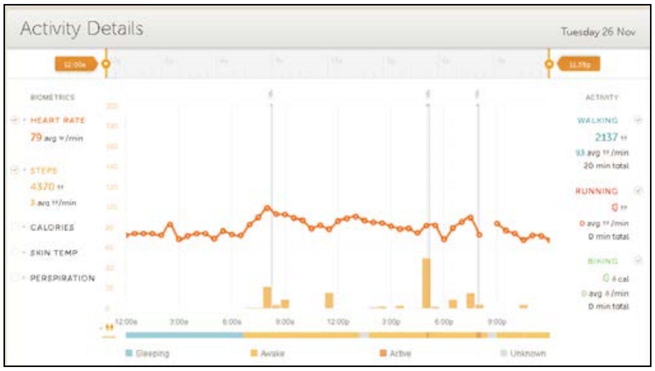

Basis (PC)

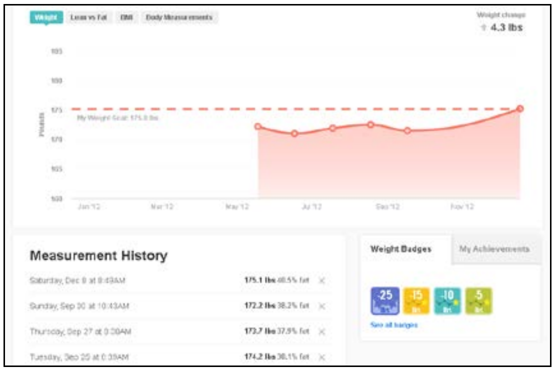

Fitbit (PC)

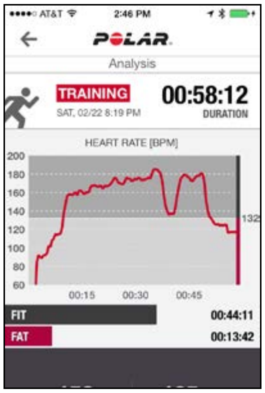

Polar (iOS)

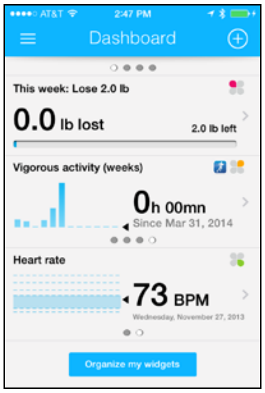

Withings (iOS)

Please note that Fitbit and Polar data required additional purchases (WiFi scale and heart rate monitor) BodyMedia and Withings also have the capability of automatically monitoring weight from WiFi scales but are not shown here

2.7. Feedback on outcome(s) of behavior

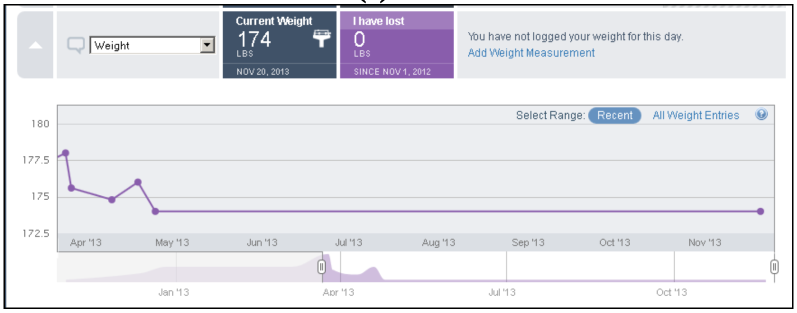

BodyMedia (PC)

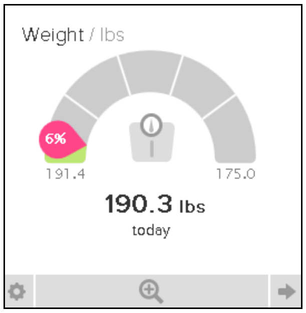

Fitbit (PC)

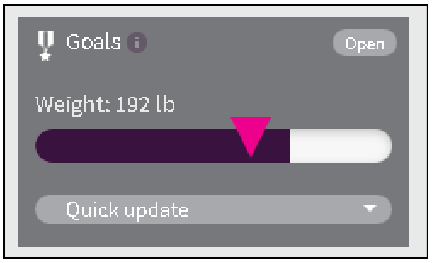

Fitbug (PC)

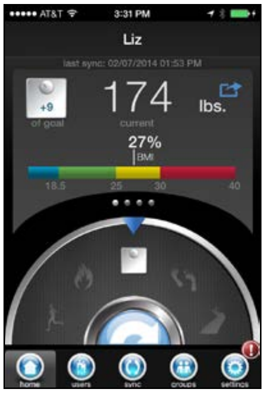

Ibitz (iOS)

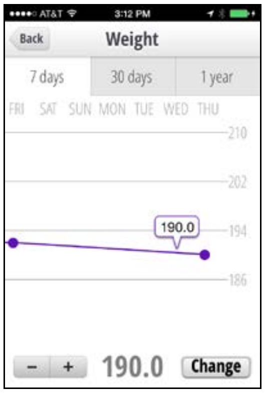

Striiv Walkathon (iOS)

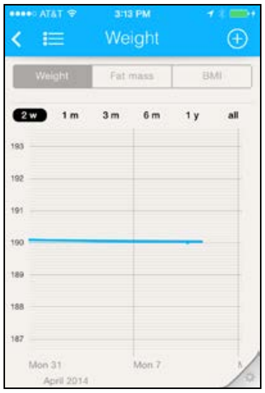

Withings (iOS)

3. Social support

3.1. Social support (unspecified)

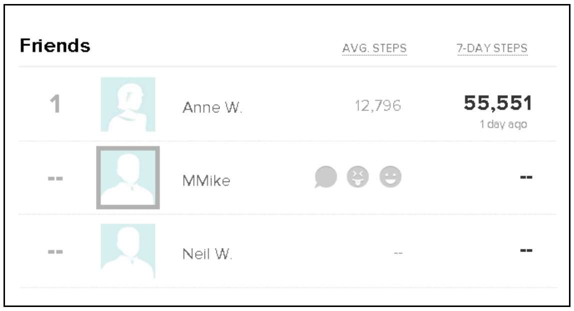

Fitbit (PC)

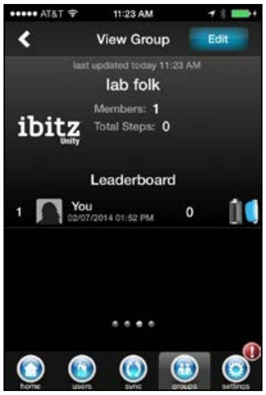

Ibitz (iOS)

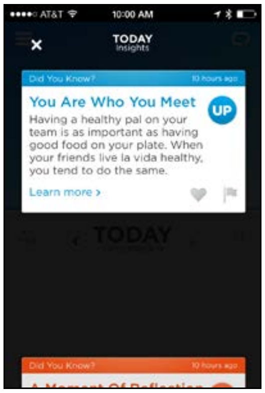

Jawbone (iOS)

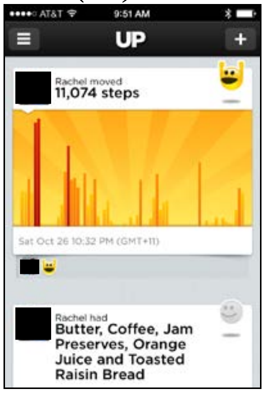

Jawbone (iOS)

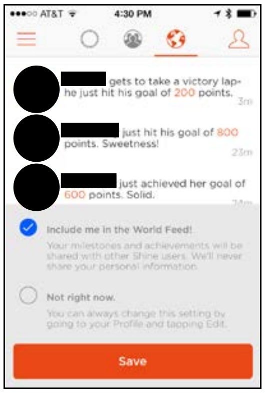

Misfit (iOS)

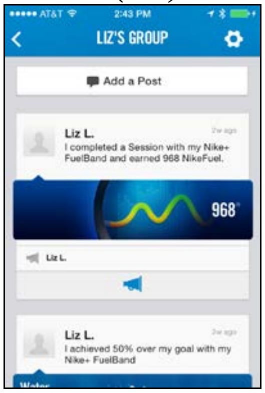

Nike+ (iOS)

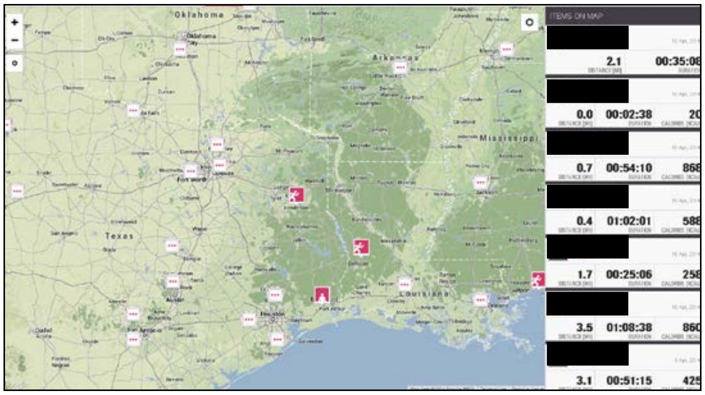

Polar (PC)

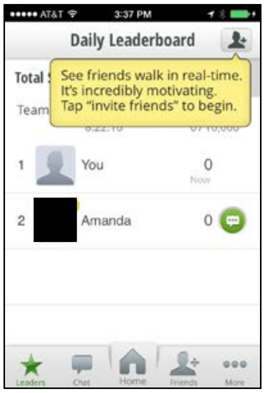

Striiv Walkathon(iOS)

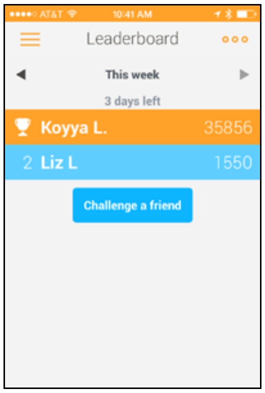

Withings (iOS)

3.2. Social support (practical)

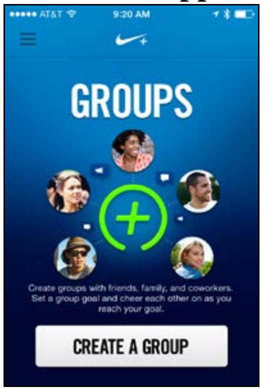

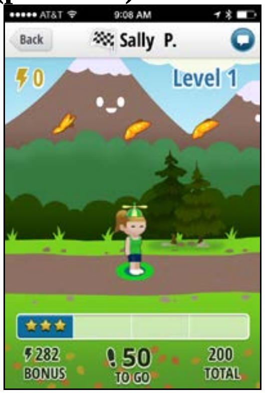

Nike+ (iOS) *Striiv Walkathon(iOS)*

3.3. Social support (emotional)

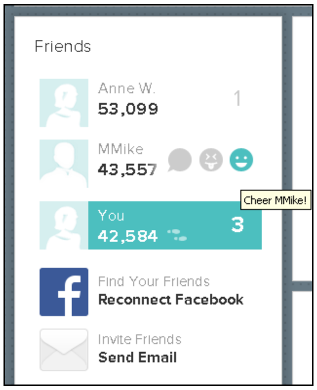

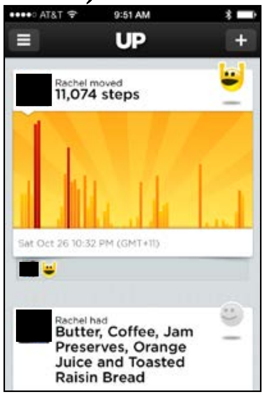

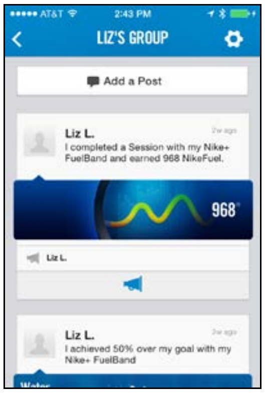

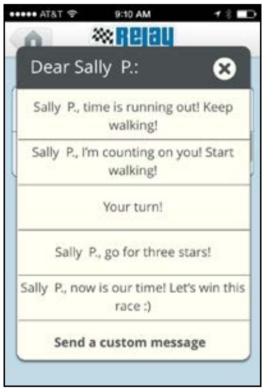

Fitbit (PC) *Jawbone (iOS)* *Nike+ (iOS)* *Striiv Walkathon(iOS)*

<u>4. Shaping knowledge</u>

4.1. Instruction on how to perform the behavior

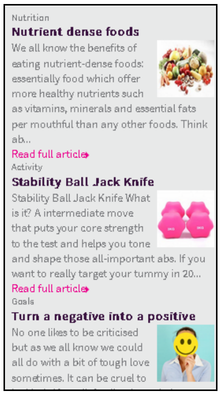

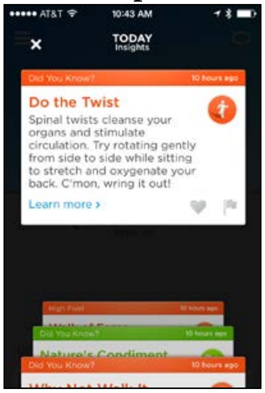

Fitbug (PC) *Jawbone (iOS)*

4.2. Information about antecedents

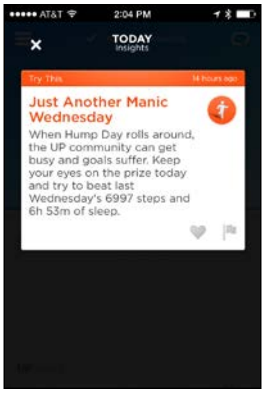

Jawbone (iOS)

<u>5. Natural consequences</u>

5.1. Information about health consequences

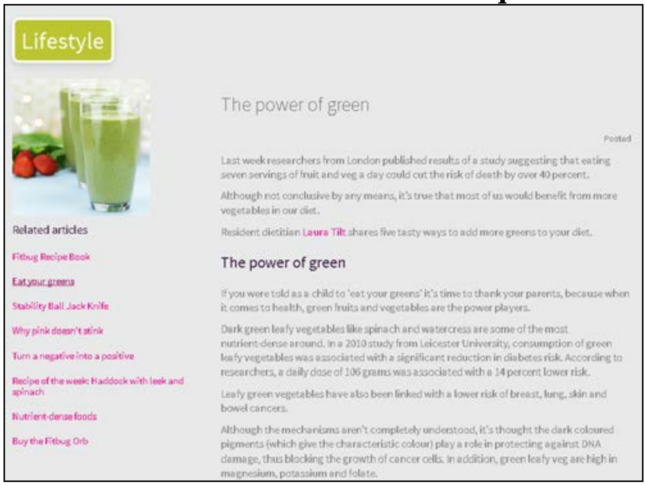

Fitbug (PC)

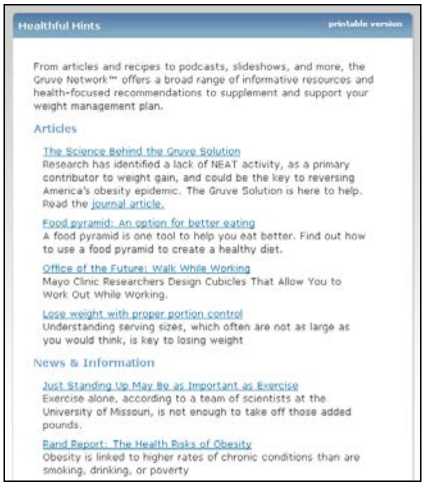

Gruve (PC)

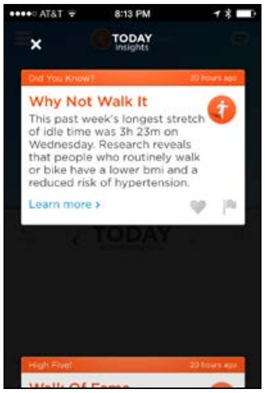

Jawbone (iOS)

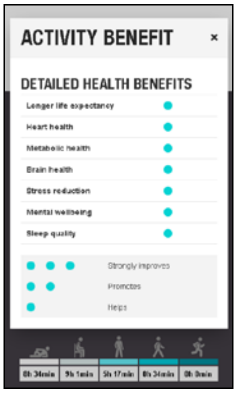

Polar (PC)

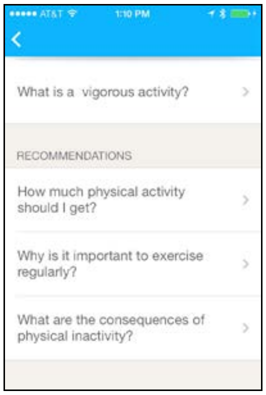

Withings (iOS)

5.3. Information about social and environmental consequences

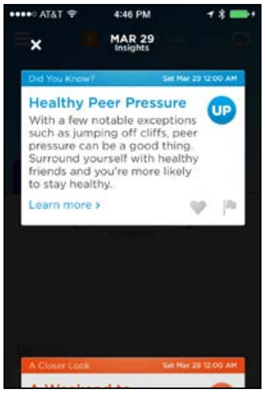

Jawbone (iOS)

5.4. Monitoring of emotional consequences

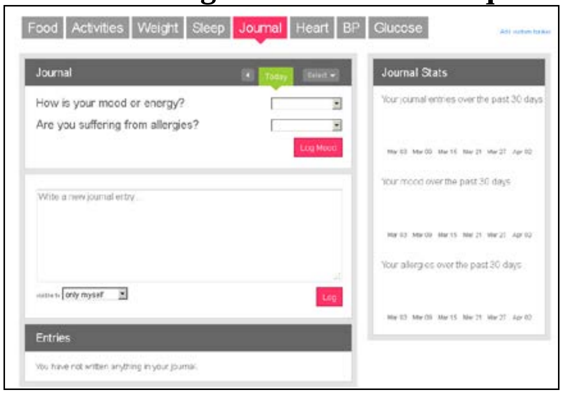

Fitbit (PC)

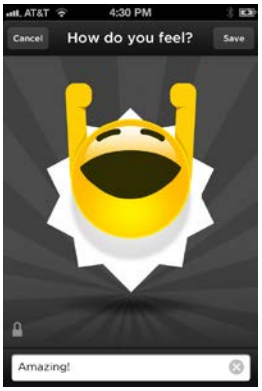

Jawbone (iOS)

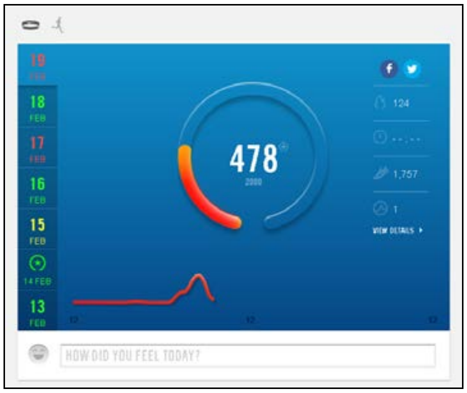

Nike+ (PC)

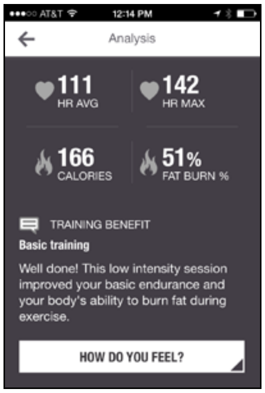

Polar (iOS)

5.6. Information about emotional consequences

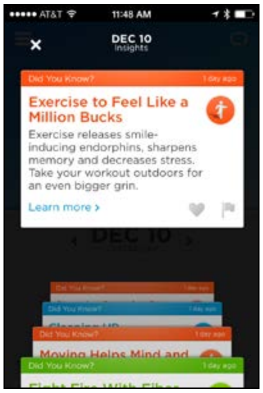

Jawbone (iOS)

6. Comparison of behavior

6.2. Social comparison

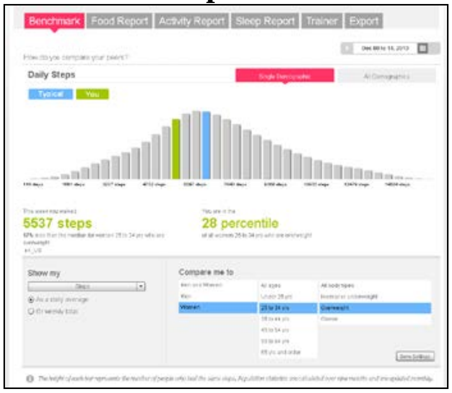

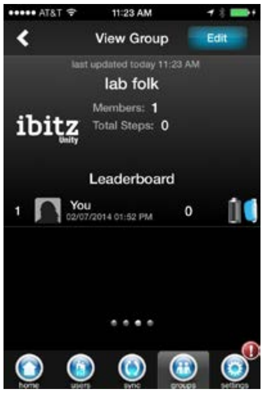

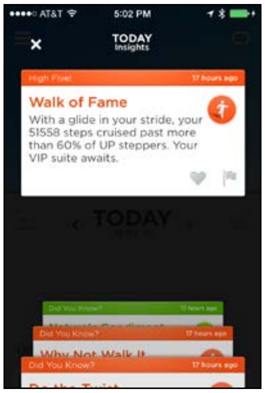

Fitbit (PC) *Ibitz (iOS)* *Jawbone (iOS)*

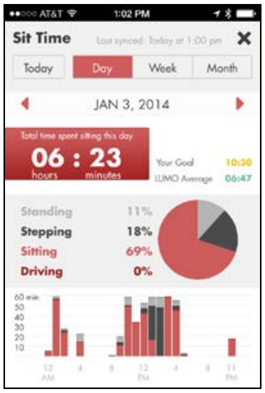

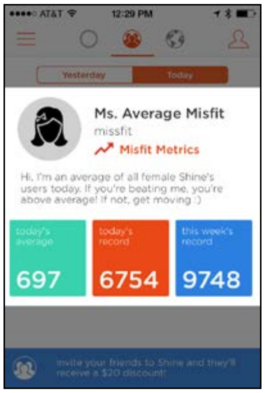

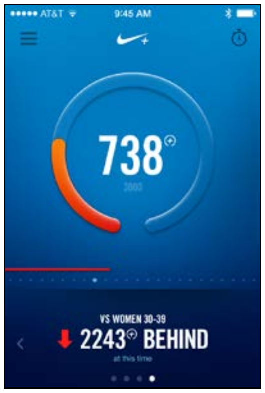

Lumo (iOS) *Misfit (iOS)* *Nike+ (iOS)*

7. Associations

7.1. Prompts/cues

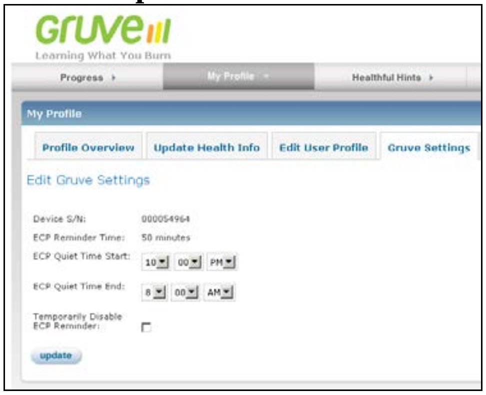

Gruve (PC)

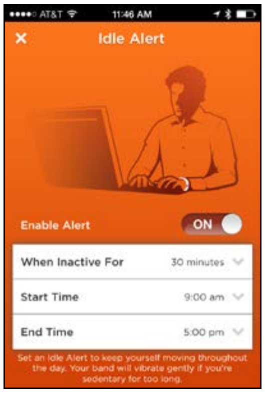

Jawbone (iOS)

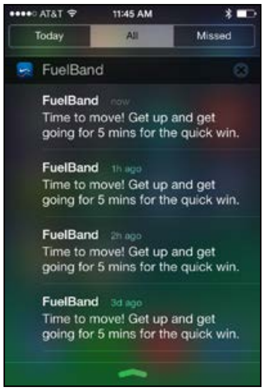

Nike+ (iOS)

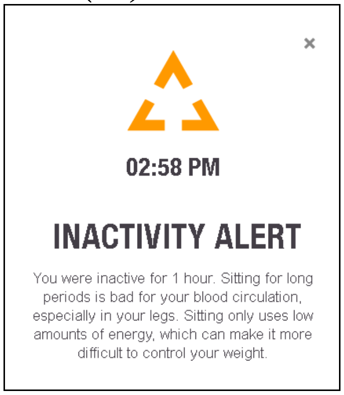

Polar (PC)

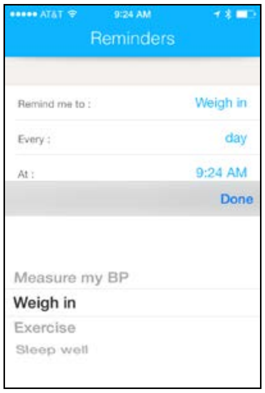

Withings (iOS)

8. Repetition and substitution

8.2. Behavior substitution

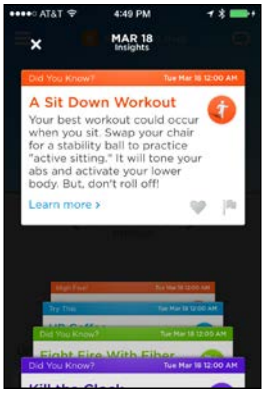

Jawbone (iOS)

8.3. Habit formation

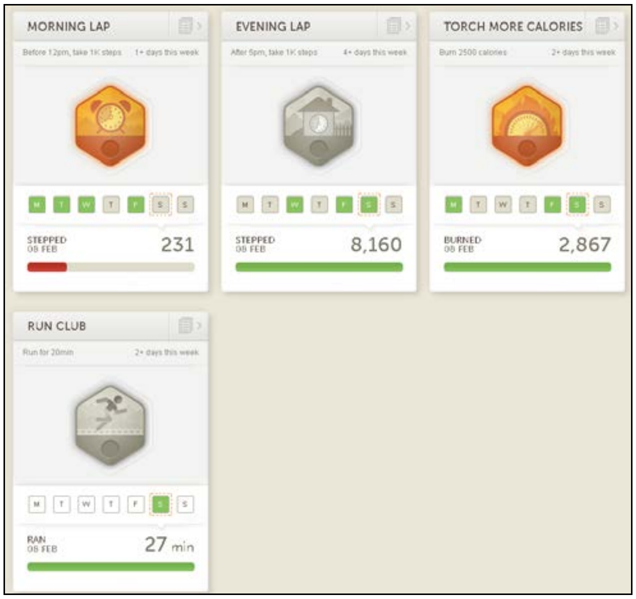

Basis (PC)

8.7. Graded tasks

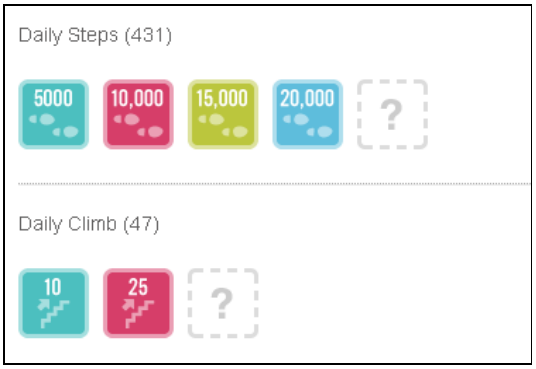

Fibit (PC)

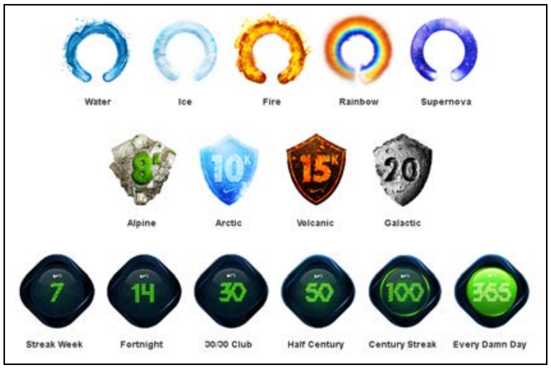

Nike+ (PC)

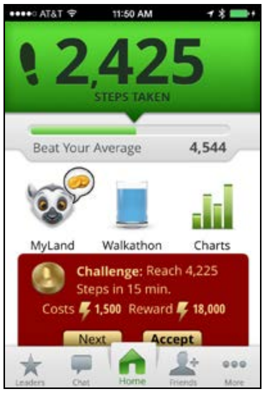

Striiv Walkathon(iOS)

9. Comparison of outcomes

9.1. Credible source

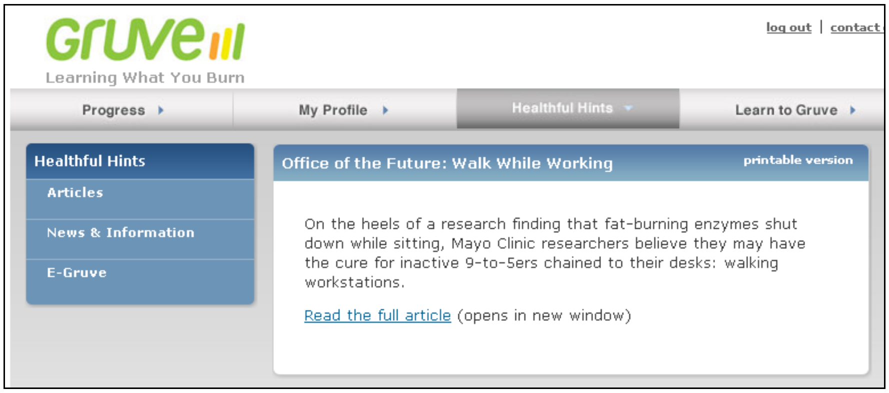

Gruve (PC)

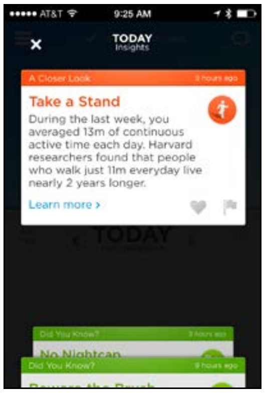

Jawbone (iOS)

10. Reward and threat

10.3. Non-specific reward

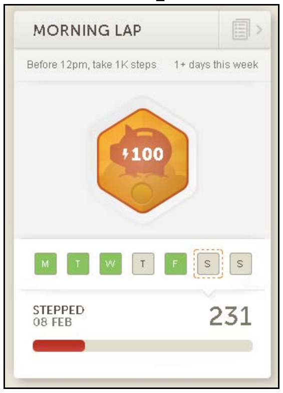

Basis (PC)

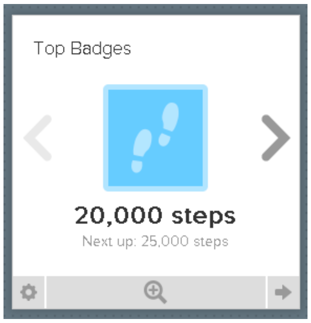

Fitbit (PC)

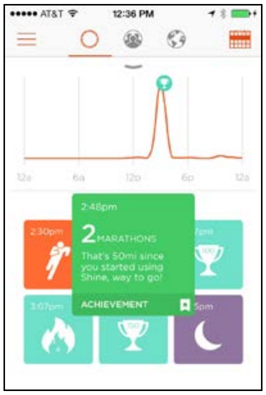

Misfit (iOS)

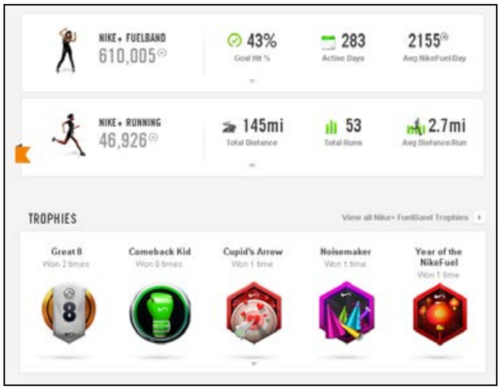

Nike+ (PC)

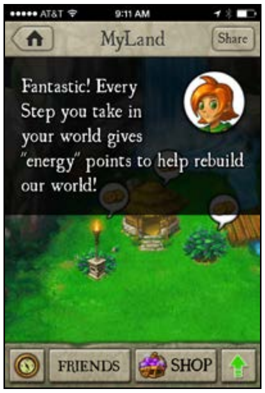

Striiv Walkathon(iOS)

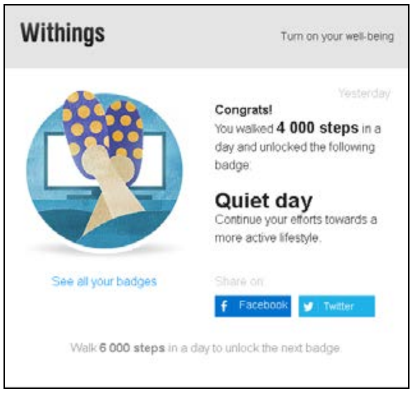

Withings (iOS, badge via email)

10.4. Social reward

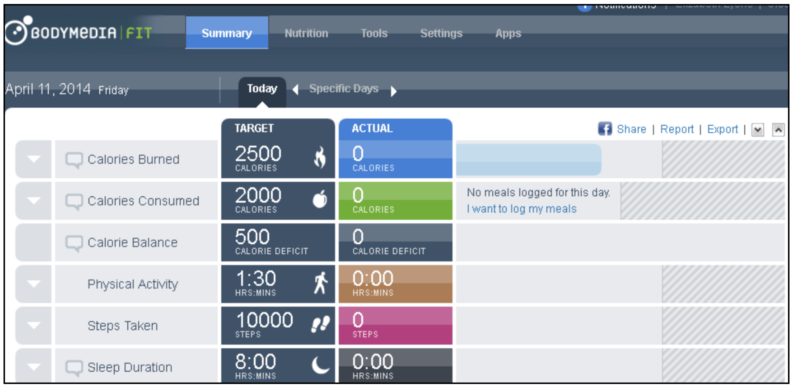

BodyMedia (PC)

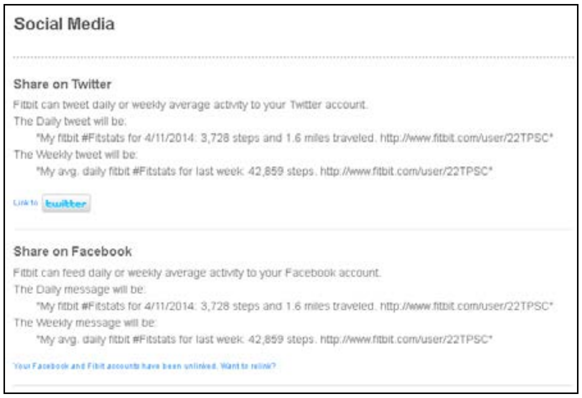

Fitbit (PC)

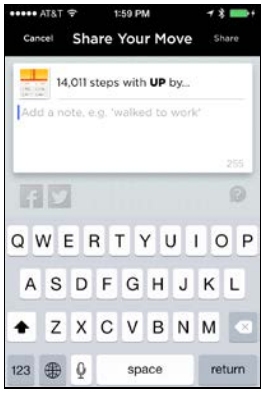

Jawbone (iOS)

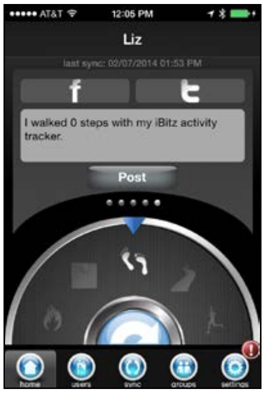

Ibitz (iOS)

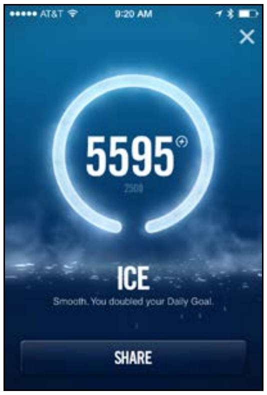

Nike+ (iOS)

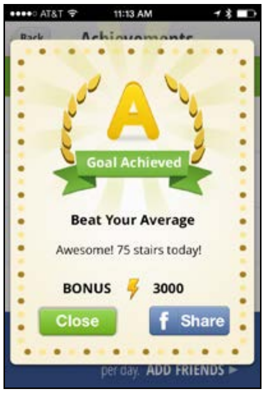

Striiv Walkathon(iOS)

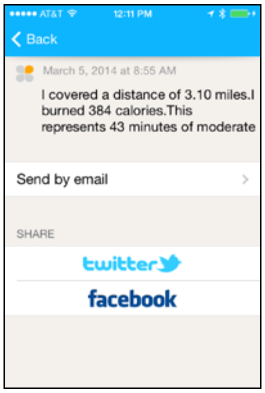

Withings (iOS)

10.10. Reward (outcome)

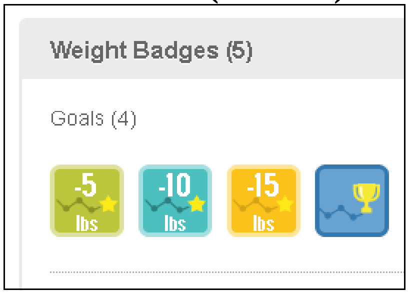

Fitbit (PC)

14. Scheduled consequences

14.6. Situation-specific reward

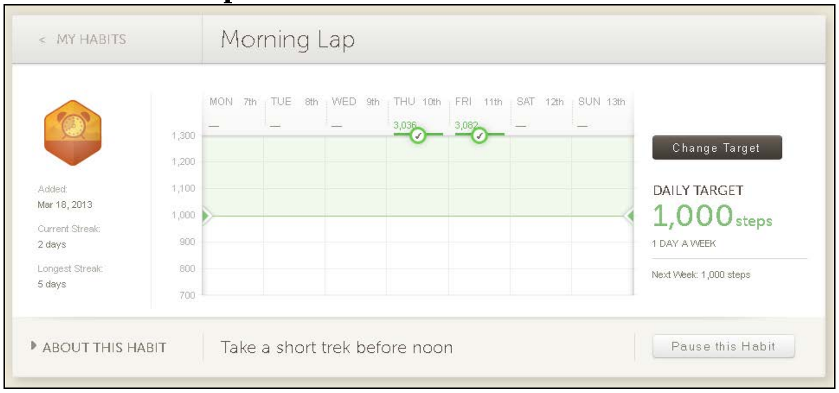

Basis (PC)

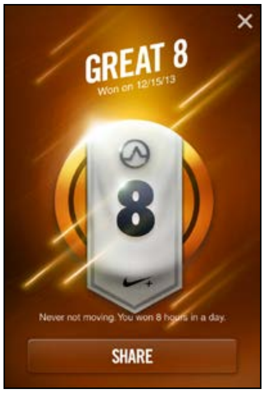

Nike+ (iOS)

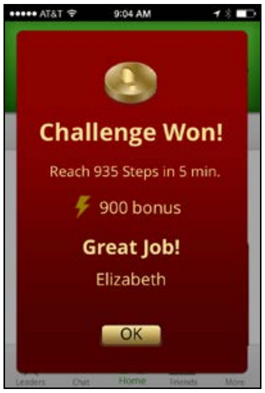

Striiv Walkathon (iOS)

14.7. Reward incompatible behavior

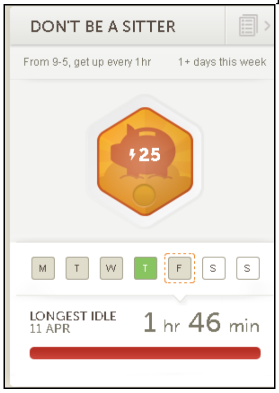

Basis (PC)

15. Self-belief

15.3. Focus on past success

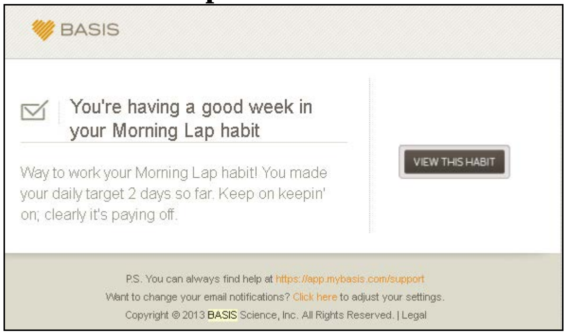

Basis (PC, notice via email)

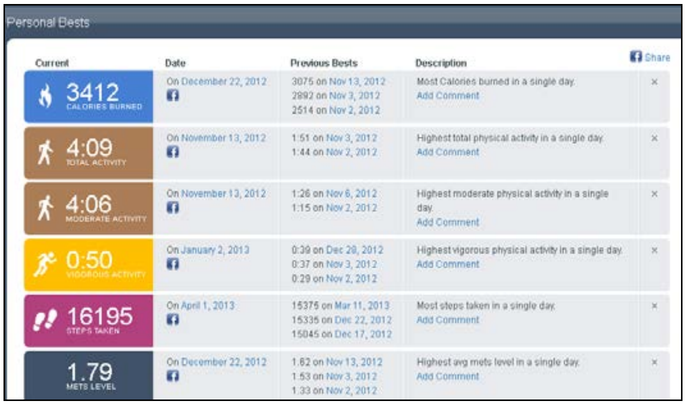

BodyMedia (PC)

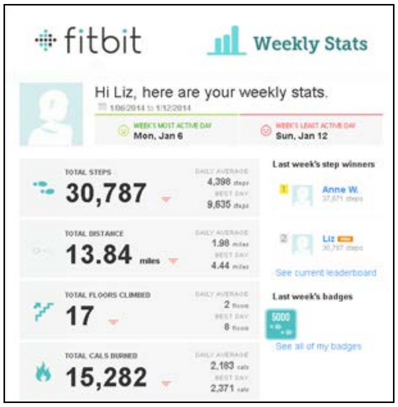

Fitbit (PC, notice via email)

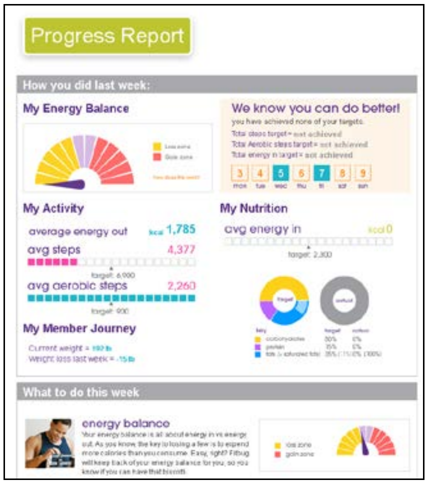

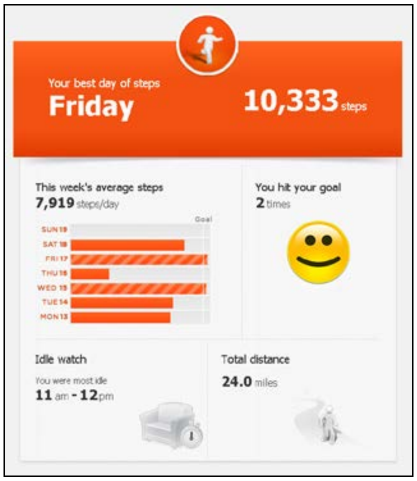

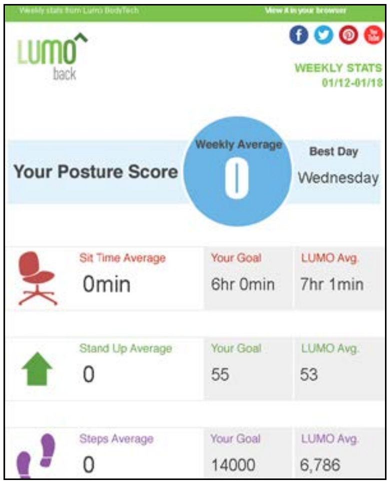

Fitbug (PC, notice via email) *Jawbone (iOS, notice via email)* *Lumo (iOS, notice via email)*

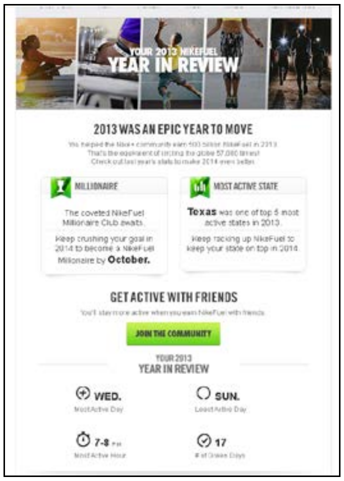

Nike+ (PC, notice via email)

Please note that not all instances that were coded are shown here; in some cases, we were not able to replicate the conditions that produced some behavior change techniques when we were taking screenshots for the purposes of this appendix.
