# Supplementary material for: Behavior Change Techniques Implemented in Electronic Lifestyle Activity Monitors: A Systematic Content Analysis
Source: J Med Internet Res. 2014 Aug 15;16(8):e192. doi: 10.2196/jmir.3469 (PMC4147713; doi:10.2196/jmir.3469)
Supplement: Supplementary file 2 [file jmir_v16i8e192_app2.pdf]

Multimedia Appendix 2. Behavior Change Techniques by Monitor

Table 1. Behavior Change Techniques from the hierarchical taxonomy

**BCT**	**Basis**	**Bodymedia**	**Fitbit**	**Fitbug**	**Gruve**	**Ibitz**	**Jawbone**	**Lumo**	**Misfit**	**Nike**	**Polar**	**Striiv**	**Withings**	**Total**
1. Goals and planning														
1.1 Goal setting (behavior)	x	x	x	x	x	x	x	x	x	x	x	X	x	13
1.2 Problem solving							x							1
1.3 Goal setting (outcome)		x	x	x	x	x	x		x				x	8
1.4 Action planning	x						x			x		*x*	x	5
1.5 Review behavior goal(s)	x	x	x			x	x	x	x	x		X	x	10
1.6 Discrepancy between current behavior and goal	x	x	x	x	x	x	x	x	x	x	x	X	x	13
1.7 Review outcome goal(s)		x	x	x		x	x		x				x	7
1.8 Behavioral contract														0
1.9 Commitment							x	x		x		*x*		4
2. Feedback and monitoring														
2.1 Monitoring of behavior by others without feedback														0
2.2 Feedback on behavior	x	x	x	x	x	x	x	x	x	x	x	x	x	13
2.3 Self-monitoring of behavior	x	x	x	x	x	x	x	x	x	x	x	x	x	13
2.4 Self-monitoring of outcome(s) of behavior		x	x	x		x	x		x			x	x	8
2.5 Monitoring of outcome(s) of behavior without feedback														0
2.6 Biofeedback	x	x	x	x							x		x	2
2.7 Feedback on outcome(s) of behavior		x	x	x		x	x		x			x	x	8
3. Social support														
3.1. Social support (unspecified)			x			x	x		x	x	x	x	x	8
3.2. Social support (practical)										x		x		2
3.3. Social support (emotional)			x				x			x		x		4
4. Shaping knowledge														
4.1 Instruction on how to perform the behavior				x			x							2

**BCT**	**Basis**	**Bodymedia**	**Fitbit**	**Fitbug**	**Gruve**	**Ibitz**	**Jawbone**	**Lumo**	**Misfit**	**Nike**	**Polar**	**Striiv**	**Withings**	**Total**
4.2 Information about antecedents							x							1
4.3 Re-attribution														0
4.4 Behavioral experiments														0
5. Natural consequences														
5.1 Information about health consequences		x		x	x		x				x		x	6
5.2 Salience of consequences														0
5.3 Information about social and environmental consequences							x							1
5.4 Monitoring of emotional consequences			x				x			x	x			4
5.5 Anticipated regret														0
5.6 Information about emotional consequences							x							1
6. Comparison of behavior														
6.1 Demonstration of the behavior														0
6.2 Social comparison			x			x	x	x	x	x	x	x		8
6.3 Information about others' approval														0
7. Associations														
7.1 Prompts/cues					x		x	x		x	x	*x*	x	7
7.2 Cue signaling reward														0
7.3 Reduce prompts/cues														0
7.4 Remove access to the reward														0
7.5 Remove aversive stimulus														0
7.6 Satiation														0
7.7 Exposure														0
7.8 Associative learning														0
8 Repetition and substitution														
8.1 Behavioral practice/rehearsal														0
8.2 Behavior substitution							x							1

**BCT**	**Basis**	**Bodymedia**	**Fitbit**	**Fitbug**	**Gruve**	**Ibitz**	**Jawbone**	**Lumo**	**Misfit**	**Nike**	**Polar**	**Striiv**	**Withings**	**Total**
8.3 Habit formation	x													1
8.4 Habit reversal														0
8.5 Overcorrection														0
8.6 Generalisation of target behavior														0
8.7 Graded tasks			x							x		*x*		3
9. Comparison of outcomes														
9.1 Credible source					x		x							2
9.2 Pros and cons														0
9.3 Comparative imagining of future outcomes														0
10. Reward and threat														
10.1 Material incentive (behavior)														0
10.2 Material reward (behavior)														0
10.3 Non-specific reward	x		x						x	x		x	x	6
10.4 Social reward		x	x			x	x	x		x		x	x	8
10.5 Social incentive														0
10.6 Non-specific incentive														0
10.7 Self-incentive														0
10.8 Incentive (outcome)														0
10.9 Self-reward														0
10.10 Reward (outcome)			x											1
10.11 Future punishment														0
11. Regulation														
11.1 Pharmacological support														0
11.2 Reduce negative emotions														0
11.3 Conserving mental resources														0
11.4 Paradoxical instructions														0
12. Antecedents														

**BCT**	**Basis**	**Bodymedia**	**Fitbit**	**Fitbug**	**Gruve**	**Ibitz**	**Jawbone**	**Lumo**	**Misfit**	**Nike**	**Polar**	**Striiv**	**Withings**	**Total**
12.1 Restructuring the physical environment														0
12.2 Restructuring the social environment														0
12.3 Avoidance/reducing exposure to cues for the behavior														0
12.4 Distraction														0
12.5 Adding objects to the environment	x	x	x	x	x	x	x	x	x	x	x	x	x	13
12.6 Body changes														0
13. Identity														
13.1 Identification of the self as role model														0
13.2 Framing/reframing														0
13.3 Incompatible beliefs														0
13.4 Valued self-identity														0
13.5 Identity associated with changed behavior														0
14. Scheduled consequences														
14.1 Behavior cost														0
14.2 Punishment														0
14.3 Remove reward														0
14.4 Reward approximation														0
14.5 Rewarding completion														0
14.6 Situation-specific reward	x									x		*x*		3
14.7 Reward incompatible behavior	x													1
14.8 Reward alternative behavior														0
14.9 Reduce reward frequency														0
14.10 Remove punishment														0
15. Self-belief														
15.1 Verbal persuasion about capability														0
15.2 Mental rehearsal of successful performance														0
15.3 Focus on past success	x	x	x	x			x	x		x				7

BCT	Basis	Bodymedia	Fitbit	Fitbug	Gruve	Ibitz	Jawbone	Lumo	Misfit	Nike	Polar	Striiv	Withings	Total
15.4 Self-talk														0
16. Covert learning														
16.1 Imaginary punishment														0
16.2 Imaginary reward														0
16.3 Vicarious consequences														0
Total techniques	13	14	20	13	9	13	27	11	13	19	11	14 / *16*	17	

Shaded lines indicate that additional purchases are required
Capital X marks in the Striiv column refer to capabilities only available in the Striiv Activity Tracker app, whereas a lowercase x in italics refers to capabilities only available in the Striiv Walkathon and Fitness Tracker app. Standard lowercase x marks indicate that the function is available in both apps. The numbers of total techniques for these apps are represented with lowercase numbers (Striiv Activity Tracker) and italics (Striiv Walkathon and Fitness Tracker).

Further specifications for coding of several techniques used by coders, Table 1:
1.1 Goal setting (behavior): Daily and more distal goals were counted. If the only goal-setting was in small challenges (e.g., step as much as possible for one minute), it was not counted.
1.3 Goal setting (outcome): the outcome was weight.
1.4 Action planning: badges and challenges that encouraged certain behaviors and/or activity on particular dates/times of day were counted.
1.5 Review behavior goals: the ability to change activity goals was counted. Several apps also suggested that users review their goals.
1.9 Commitment: asking users to agree to a challenging, small goal was counted.
2.3 Self-monitoring of behavior: all automatic monitoring by the monitors was considered self-monitoring.
2.6 Biofeedback: hatched squares indicate that biofeedback is available within the app if an additional sensor is purchased (e.g., scale, heart rate monitor, blood pressure monitor).
3.1 Social support (unspecified): the ability to friend or follow other users, create teams or groups, or otherwise interact with others were counted.

3.2 Social support (practical): the ability to walk/exercise simultaneously with others or interact with others during an activity session was counted.
3.3 Social support (emotional): the ability to send and receive messages, emoticons, cheers, etc. was counted.
4.1 Instruction on how to perform the behavior: only instructions related to performance of exercise activities were counted.
7.1 Prompts/cues: push notifications (notifications that cause smartphones to vibrate/chime and display a message) and monitor-based cues (vibrations, flashing lights, messages on the display) were counted.
8.7 Graded tasks: though theoretically any monitor/app that included user-entered goals could provide graded tasks, this technique was only coded as present when graded tasks were explicitly encouraged by the app. For example, different badges available for increasing levels of difficulty/effort were counted.
9.1 Credible source: links to credible websites (Centers for Disease Control, various University-based sites) were counted.
10.3 Non-specific reward: virtual rewards such as badges, trophies, and achievements were counted as non-specific rewards rather than imaginary rewards.
10.4 Social reward: ability to share progress/goal achievement to social networks was counted. If positive social interactions occurred within the app (for example, amongst members of a group or team), the interactions were coded as social support.
10.10 Reward (outcome): badges related to weight loss were counted.
12.5 Adding objects to the environment: activity monitors were considered additions to the environment.
14.6 Situation-specific reward: badges/trophies/achievements related to specific situations, such as reaching one's daily goal on Valentine's Day or achieving a certain step count within thirty minutes, were counted.
14.7 Reward incompatible behavior: badges for breaking up sedentary behavior bouts were counted.
15.3 Focus on past success: to be counted as this technique, rather than just as feedback, an app must have provided some kind of special emphasis on comparing past behavior to present behavior. Emails that provided an overview of weekly, monthly, and in some cases yearly activity and goal achievement were counted.

Table 2. Fidelity of BCT implementation to Social Cognitive Theory principles

**Technique and recommendation**	**Basis**	**Bodymedia**	**Fitbit**	**Fitbug**	**Gruve**	**Ibitz**	**Jawbone**	**Lumo**	**Misfit**	**Nike**	**Polar**	**Striiv**	**Withings**	**Total**
Goal-setting														
Specific	x	x	x	x	x	x	x	x	x	x	x	x	x	13
Measureable	x	x	x	x	x	x	x	x	x	x	x	x	x	13
Moderately challenging	x	x	x	x	x	x	x	x	x	x		x	x	13
Long-term goals broken into short-term goals		x	x	x	x	x							x	5
Easier goals successfully accomplished before attempting more difficult ones			x							x		x		3
Self-monitoring														
Conducted regularly	x	x	x	x	x	x	x	x	x	x	x	x	x	13
Conducted close in time to target activity	x	x	x	x	x	x	x	x	x	x	x	x	x	13
Track precise information	x	x	x	x	x	x	x	x	x	x	x	x	x	13
Track personally valued information	x	x	x	x							x		x	6
Emphasize performance successes	x	x	x	x			x	x		x		x	x	9
Focus on behavior modifiable by deliberate effort	x	x	x	x	x	x	x	x	x	x	x	x	x	13
Feedback														
Specific	x	x	x	x	x	x	x	x	x	x	x	x	x	13
Give a clear idea of how well the participant is doing	x	x	x	x	x	x	x	x	x	x	x	x	x	13
Compare performance to past accomplishments	x	x	x	x	x	x	x	x	x	x	x	x	x	13
Compare performance to norms of similar groups			x				x	x	x	x				5
Compare performance to precise goals	x	x	x	x	x	x	x	x	x	x	x	x	x	13

Further specifications for coding of several techniques used by coders, Table 2:

Goal-setting

Specific: clear numerical step or activity goals for a particular time period were counted.

Measureable: numerical goals were counted.

Moderately challenging: if goals could be adjusted by the user, they were counted as being capable of being moderately challenging. Also, if goals were pre-set based on an initial week of baseline wear (Gruve, Fitbug) and set to be moderately challenging, they were

included. It was unclear how the daily goals were calculated by the Polar app, but the app suggests that they are based on age and gender. We considered the goal provided to a 33 year old woman to be more than moderately challenging (to reach 100%, it was suggested that we could accumulate 7 hours 20 minutes of low intensity, 2 hours 12 minutes of medium intensity, or 58 minutes of high intensity activity – or a combination).
Long-term goals broken into short-term goals: this was counted if both long-term and short-term goals existed. Many apps only gave daily goals for activity. Long-term goals were typically weight-related and were not always specific/numeric.
Easier goals successfully accomplished before attempting more difficult ones: as with the BCT "graded tasks" in Table 1, this consisted of instances in which accomplishing easier goals and moving on to more difficult goals was encouraged. In all three instances, this progression was encouraged in the form of progressive badges/trophies/achievements.

Self-monitoring
Conducted regularly: all monitors were capable of continuously measuring lifestyle activity.
Conducted close in time to target activity: all monitors continuously measured activity.
Track precise information: all monitors provided numerical output for their measured behaviors/outcomes.
Track personally valued information: if trackers could be individualized, it was counted. For example, some apps provided the opportunity to track additional health indicators such as blood pressure, fitness, and waist circumference.
Emphasize performance successes: Emails, notifications, and written encouragement were counted.
Focus on behavior modifiable by deliberate effort: all monitors measured changeable physical activity.

Feedback
Specific: all monitors provided feedback in the form of specific step counts, etc.
Give a clear idea of how the participant is doing: charts and numerical totals were counted.
Compare performance to past accomplishments: chart-based and numerical comparisons to previous days/weeks/etc. were counted.
Compare performance to norms of similar groups: explicit comparison to similar groups (e.g., women aged 30-35, sedentary men, the average user of the monitor) were counted.
Compare performance to precise goals: monitors with specific goals were counted, as all presented activity totals in relation to stated goals.
