# Supplementary material for: Behavior Change Techniques Implemented in Electronic Lifestyle Activity Monitors: A Systematic Content Analysis
Source: J Med Internet Res. 2014 Aug 15;16(8):e192. doi: 10.2196/jmir.3469 (PMC4147713; doi:10.2196/jmir.3469)
Supplement: Supplementary file 3 [file jmir_v16i8e192_app3.pdf]

Multimedia Appendix 3. Potentially effective techniques by monitor system.

BCT	Basis	Bodymedia	Fitbit	Fitbug	Gruve	Ibitz	Jawbone	Lumo	Misfit	Nike	Polar	Striiv	Withings
Goal setting (behavior)	■	■	■	■	■	■	■	■	■	■	■	■	■
Problem solving							■						
Goal setting (outcome)		■	■	■	■	■	■		■				■
Action planning	■						■			■		■	■
Review behavior goal(s)	■	■	■			■	■	■	■	■		■	■
Commitment							■	■		■		■	
Feedback on behavior	■	■	■	■	■	■	■	■	■	■	■	■	■
Self-monitoring of behavior	■	■	■	■	■	■	■	■	■	■	■	■	■
Social support (any kind)			■			■	■		■	■	■	■	■
Instruction on how to perform the behavior				■			■						
Information about consequences		■		■	■		■				■		■
Social comparison			■			■	■	■	■	■	■	■	
Behavioral practice/rehearsal													
Rewards (any kind)	■	■	■			■	■	■	■	■		■	■
Total	6	7	8	6	5	8	13	7	8	9	6	9	9
